# Microglial cell loss after ischemic stroke favors brain neutrophil accumulation

**DOI:** 10.1007/s00401-018-1954-4

**Published:** 2018-12-22

**Authors:** Amaia Otxoa-de-Amezaga, Francesc Miró-Mur, Jordi Pedragosa, Mattia Gallizioli, Carles Justicia, Núria Gaja-Capdevila, Francisca Ruíz-Jaen, Angélica Salas-Perdomo, Anna Bosch, Maria Calvo, Leonardo Márquez-Kisinousky, Adam Denes, Matthias Gunzer, Anna M. Planas

**Affiliations:** 1 Department of Brain Ischemia and Neurodegeneration, Institut d’Investigacions Biomèdiques de Barcelona (IIBB)-Consejo Superior de Investigaciones Científicas (CSIC), Rossello 161 planta 6, Barcelona, 08036 España; 2 Area of Neuroscience, Institut d’Investigacions Biomèdiques August Pi i Sunyer (IDIBAPS), Barcelona, Spain; 3 Scientific and Technological Centers, Universitat de Barcelona (CCiTUB) Campus Medicina Clínic, Barcelona, Spain; 4 Laboratory of Neuroimmunology, Institute of Experimental Medicine, Hungarian Academy of Sciences, Budapest, Hungary; 5 Institute for Experimental Immunology and Imaging, University Hospital, University Duisburg-Essen, Essen, Germany

**Keywords:** Microglia, Neutrophils, Brain ischemia, Mouse, Human, Phagocytosis, Colony stimulating factor 1 receptor

## Abstract

Stroke attracts neutrophils to the injured brain tissue where they can damage the integrity of the blood–brain barrier and exacerbate the lesion. However, the mechanisms involved in neutrophil transmigration, location and accumulation in the ischemic brain are not fully elucidated. Neutrophils can reach the perivascular spaces of brain vessels after crossing the endothelial cell layer and endothelial basal lamina of post-capillary venules, or migrating from the leptomeninges following pial vessel extravasation and/or a suggested translocation from the skull bone marrow. Based on previous observations of microglia phagocytosing neutrophils recruited to the ischemic brain lesion, we hypothesized that microglial cells might control neutrophil accumulation in the injured brain. We studied a model of permanent occlusion of the middle cerebral artery in mice, including microglia- and neutrophil-reporter mice. Using various in vitro and in vivo strategies to impair microglial function or to eliminate microglia by targeting colony stimulating factor 1 receptor (CSF1R), this study demonstrates that microglial phagocytosis of neutrophils has fundamental consequences for the ischemic tissue. We found that reactive microglia engulf neutrophils at the periphery of the ischemic lesion, whereas local microglial cell loss and dystrophy occurring in the ischemic core are associated with the accumulation of neutrophils first in perivascular spaces and later in the parenchyma. Accordingly, microglia depletion by long-term treatment with a CSF1R inhibitor increased the numbers of neutrophils and enlarged the ischemic lesion. Hence, microglial phagocytic function sets a critical line of defense against the vascular and tissue damaging capacity of neutrophils in brain ischemia.

**Electronic supplementary material:**

The online version of this article (10.1007/s00401-018-1954-4) contains supplementary material, which is available to authorized users.

## Introduction

Neutrophil infiltration under conditions of sterile inflammation can contribute to tissue injury. Neutrophils are transiently detected in the brain after stroke since they are rapidly attracted to the injured brain peaking between 1 and 3 days post-ischemia [10, 23, 24, 51]. Compelling evidence suggests that neutrophils are contributors to tissue damage after ischemic stroke [35, 44, 51, 61, 64], in spite of the fact that diverse experimental strategies inhibiting neutrophil activation or depleting neutrophils provided conflicting results [16, 61]. Likely, the differences between experimental studies depend on the efficacy and potential side effects of the diverse neutrophil depleting or inhibiting strategies, status of capillary reperfusion, lesion severity, and integrity of the blood–brain barrier (BBB). Moreover, several aspects of neutrophil infiltration after acute ischemic brain damage remain controversial. Neutrophils accumulate in perivascular spaces in murine and human strokes [17, 55]. The presence of neutrophils in the brain parenchyma has been reported in rodent models of permanent ischemia [23, 51, 55], but it is more controversial in experimental models of transient ischemia [17, 64]. Several studies reported the presence of neutrophils in the brain parenchyma in post-mortem samples of patients deceased between day 1 and 5 [55], or 3 days after stroke onset but not at other time points [56, 76]. In other studies, neutrophils were not detected in the brain parenchyma of stroke patients [17]. Therefore, the molecular determinants underlying perivascular neutrophil accumulation and the conditions facilitating the potential access of neutrophils to the brain parenchyma need further clarification.

The observation that microglia phagocytose neutrophils in the ischemic brain [50–52] led us to hypothesize that microglia function may be critical to explain neutrophil accumulation in the injured brain tissue. Microglial cells react to brain ischemia in different ways depending on the regional location and temporal course of the lesion. Microglial cells are vulnerable to ischemia and previous reports showed death of microglia after oxygen and glucose deprivation in tissue slices [18] and cell cultures [41, 73]. In addition, microglial reduction has been reported after transient MCAo [43], and microglial dysfunction and loss was detected in classical neuropathological studies of brain ischemia in rodents and primates [3, 4]. Classical histopathological studies have shown long-lasting microgliosis surrounding the infarction several days after ischemic stroke onset. However, the progression of this reaction from the very acute phase of stroke is less precisely determined mainly due to the fact that microglia and infiltrating macrophages show many common features and markers leading to the frequent terminology of microglia/macrophages to describe the mononuclear myeloid cell reaction that follows stroke. Microglia have a unique transcriptomic signature distinguishable from that of macrophages or monocytes [7, 32]. Therefore, reactive microglia and infiltrating macrophages likely play different functions in the injured brain tissue. Current developments allow the distinction between these cells with antibodies against more specific microglia markers [1, 7], availability of fluorescent reporter mice [74], or transfer of fluorescent reporter leukocytes [46]. By exploiting some of these novel experimental possibilities, we investigated the neutrophil–microglia crosstalk after brain ischemia. The results show that microglial cells effectively remove brain-infiltrating neutrophils, hence microglia dysfunction or death is associated with neutrophil accumulation into the injured brain tissue.

## Materials and methods

### Animals

We used adult male mice on the C57BL/6 background. Mice expressing tamoxifen-inducible Cre recombinase under the direction of the Cx3cr1 promoter in the mononuclear phagocyte system (Cx3cr1^cre/ERT2^) [74] (#020940 JAX^®^Mice) were crossed with either Ai_9_ mice harboring a loxP-flanked STOP cassette that prevents transcription of the red fluorescent protein tdTomato (tdT) (B6.Cg-Gt(ROSA) 26Sortm9 (CAG-tdTomato)Hze/J (#007909 JAX^®^Mice) [42], or colony stimulating factor 1 receptor (CSF1R)^+/flox^ mice (B6.Cg-*Csf1r*^tm1Jwp^/J, #021212 JAX^®^Mice). We used heterozygous Catchup^IVM^ mice expressing tdT in Ly6G^+/−^ neutrophils [26]. Homozygous Catchup^IVM^ (Ly6G^−/−^) mice were crossed with Cx3Cr1^gfp/gfp^ mice to obtain double heterozygous mice with red fluorescent neutrophils and green fluorescent microglia [50, 75]. We also obtained cells from DsRed mice constitutively expressing the red fluorescent protein DsRed under the control of the actin promoter [8]. Wild-type mice were obtained from a commercial source (Janvier, France). Mice were maintained in the animal house of the School of Medicine of the University of Barcelona under controlled SPF conditions. Animal work was conducted with the approval of the ethical committee of the University of Barcelona (CEEA) and the *Direcció General de Polítiques Ambientals i Medi Natural, Departament de Territori i Sostenibilitat de la Generalitat de Catalunya*. Studies complied with the “Principles of laboratory animal care” (NIH publication No. 86-23, revised 1985), and the Spanish National law (Real Decreto 53/2013).

### Stroke patients

The brains of six patients suffering from acute ischemic stroke who died between 1 and 6 days after stroke onset at the Stroke Unit of the Hospital Clinic of Barcelona were used after obtaining written consent from their relatives or legal representatives for tissue removal after death at the Neurological Tissue Bank of the Biobank-Hospital Clinic-Institut d’Investigacions Biomèdiques August Pi i Sunyer (IDIBAPS). The Ethics Committee of this Hospital approved the study. Online Resource 1 shows a summary of patient characteristics. The elapsed time from death to autopsy was 2–8 h. An expert neuropathologist dissected the ischemic core, periphery, and a portion of non-ischemic tissue (control) obtained from a region distant to infarction, as described [55]. Samples were embedded in OCT and immediately frozen in liquid nitrogen for sectioning at 5 µm in a cryostat.

### Generation of chimeric mice

The bone marrow of transgenic DsRed mice [46] was used to generate chimeric mice, as reported [37]. In brief, recipient adult (2-month old) wild-type mice received three intraperitoneal injections of the chemotherapeutic agent busulfan (30 mg/g body weight) 7, 5 and 3 days prior to transfer via the tail vein of five million bone marrow cells from DsRed donor mice. Mice were used 8 weeks after grafting and reconstitution was assessed by flow cytometry analysis.

### Drug treatments

To impair microglial function, mice received a daily oral administration by gavage of the CSF1R inhibitor GW2580 [12] (75 mg/kg body weight in a volume of 0.2 mL) (#S8042, Selleckchem) for 4 days, which is a dosing regimen that does not challenge microglial survival [54]. Treatment controls received the same volume of the vehicle (0.5% hydroxypropylcellulose, 0.1% Tween-80). Treatment started 2 h prior to induction of ischemia, it was randomly allocated, and was administered in a blinded fashion.

For microglia depletion, mice received the CSF1R inhibitor PLX5622 (Plexxikon) following previously reported protocols [15, 33, 69]. The inhibitor was mixed into AIN-76A standard chow at 1200 ppm (Brogaarden, Denmark). Mice (8-week-old) received the diet ad libitum for 3 weeks prior to induction of ischemia and the diet was maintained until the mice were killed. Treatment controls received AIN-76A diet for the same period of time. Both diets were given in parallel in groups of five animals per cage.

### Brain ischemia

Surgery was carried out under isoflurane anaesthesia and mice received analgesia (buprenorphine, 140 µL of a 0.015 mg/mL solution, via s.c.). Permanent occlusion of the middle cerebral artery (MCAo) was induced by coagulation of the distal portion of the right MCA together with ligation of the ipsilateral common carotid artery. This experimental model induces a focal cortical lesion in the ipsilateral hemisphere.

### Lesion volume

A subset of mice receiving the above diets (control or PLX5622) was used to study the volume of the lesion 1 day after induction of ischemia by T2w MRI in a 7.0 T BioSpec 70/30 horizontal animal scanner (Bruker BioSpin, Ettlingen, Germany), as reported [13]. Sample size was calculated using G*power 3.1 software (University of Dusseldorf) with an alpha level of 0.05, statistical power of 0.95, and estimating a size effect of 1.8 based on SD of previous results from our laboratory and published data on the effect of microglia depletion on infarct volume in other stroke models [65]. One mouse died (control diet), and one mouse was excluded (PLX5622 diet) due to surgical problems.

### In vivo BrdU incorporation

Bromodeoxyuridine (BrdU) (10 mg/mL) (#550891, BS Pharmingen) was daily injected (150 μL) via i.p. into mice starting 1 day after MCAo until day 4. One-hour after the last BrdU administration mice were killed and processed for immunofluorescence. BrdU was detected in brain tissue sections using a rat monoclonal FITC-anti-BrdU antibody (1:50, #ab74545, Abcam, Cambridge, UK) [46].

### Flow cytometry

Mouse blood and brain tissue were processed for flow cytometry as described [46]. Fc receptors were blocked by previous incubation for 10 min with CD16/CD32 (clone 2.4G2, BD Pharmingen) in FACS buffer (PBS, 2 mM EDTA, 2% FBS) at 4 °C. Live/dead Aqua cell stain (Molecular Probe, Invitrogen) was used to determine the viability of cells. Cells were incubated with the following mix of primary antibodies: CD11b (clone M1/70, APC-Cy7, BD Pharmingen), CD45 (clone 30-F11, Brilliant Violet 786, BD Horizon), Ly6G (clone 1A8, PE-Cy7, BD Pharmingen), F4/80 (clone BM8, Brilliant Violet 605, Biolegend), CD115 (clone AFS98, APC, Biolegend), CD3 (clone 17A2, violetFluor 450, Tonbo Biosciences), CD45R (clone RA3-6B2, Alexa fluor 488), Ly6C (clone HK1.4, eFluor 450, eBioScience), CD161 (NK1.1, clone PK136; PerCP/Cy5.5, Tonbo Biosciences) and CD335 (NKp46, clone 29A1.4, PerCP/Cy5.5, BD Pharmingen). Data was acquired in a BD LSRII cytometer using the FacsDiva software (BD Biosciences, San Jose, CA, USA). Data analyses were performed with FlowJo software (version X, FlowJo LLC, Ashland, OR, USA).

### Adult microglia culture

Microglia cells from adult mice (9–14 weeks old) were isolated and cultured using immunomagnetic separation (Miltenyi Biotec, Germany). Mice were perfused via the left ventricle with 60 mL of cold saline and collected in Hanks’ balanced salt solution (HBSS) buffer without calcium/magnesium (#14175-05; Life Technologies). The brain tissue was enzymatically dissociated using the Neural Tissue Dissociation Kit-P (#130-092-628; Miltenyi Biotec). The gentleMACS™ Dissociator with Heaters (#130-096-427; Miltenyi Biotec) was used for mechanical dissociation steps during 30 min at 37 °C. The digested tissue was filtered (70 µm) with HBSS buffer with calcium and magnesium (#14025-050; Life Technologies) and prepared for myelin removal process (Myelin Removal Beads II, #130-096-733; Miltenyi Biotec). Then, cells were magnetically labeled with CD11b microbeads (#130-093-634; Miltenyi Biotec) diluted in PBS supplemented with 0.5% BSA for 15 min in the dark in the refrigerator (2–8 °C). CD11b^+^ cells were collected using magnetic field columns (Miltenyi Biotec). Cell suspensions (35 μL) were then plated in complete medium consisting of DMEM medium (#10569010; Gibco-BRL) supplemented with 10% fetal bovine serum (FBS; Gibco-BRL) containing 40 U/mL penicillin and 40 μg/mL streptomycin (#15140122; Gibco-BRL) added as a drop in the middle of each well of a poly-l-lysine (#P4832; Sigma) pre-coated 8-well plate (µ-Slide 8 Well, IBIDI #80826). Cells were incubated for 30 min at 37 °C and then 250 µL of complete medium were carefully added to each well. Twenty-four hours later, we replaced 50% of complete medium, and we did a full medium change at day 5. The cells were maintained at 37 °C in a humidified atmosphere of 5% CO_2_ for 7 DIV.

### Human microglia culture from a stroke patient

We obtained human microglial cells from the ischemic tissue of one patient deceased 5 days after fatal stroke. Fresh brain tissue (about 500 mg) was harvested at autopsy (8 h after death) and was placed in a falcon tube with sterile cold RPMI 1640 medium (#21875-034, GIBCO). Visible meninges were removed, the tissue was cut in small pieces using a scalpel and incubated in a 0.25% trypsin–EDTA solution in PBS at RT for 30 min. Then, DMEM/F12 (#11330032; Gibco-BRL) with 20% FBS and DNase I (200 units/mL) was added (1:1), the tissue was disaggregated, centrifuged for 7 min at 250×*g* and the pellet was re-suspended in 30 mL DMEM/F12 supplemented with 10% FBS, 10% L-Cell conditioned medium obtained from the L929 cell line, and 100 U/mL penicillin/100 μg/mL streptomycin (#15140122; Gibco-BRL). Cells were seeded in poly-l-lysine coated T25 flasks, incubated in 5% CO_2_ at 37 °C and allowed to adhere. Culture medium was changed twice a week and at 7DIV the cells were scrapped and seeded in a 8-well plate (µ-Slide 8 Well, IBIDI #80826) previously coated O/N with poly-l-lysine. A time-lapse microscopy study was initiated 6 h later after addition of fresh bone marrow neutrophils. Afterwards, we fixed the cells for an immunofluorescence study with antibodies against the purinergic receptor P2Y, G-protein coupled, 12 (P2RY12) (1:200, #AS55042A, Anaspec).

### Neutrophil isolation and staining

Neutrophils were obtained from the bone marrow of adult (10–14 weeks old) mice. The bone marrow was flushed using a 25-gauge needle with RPMI 1640 (#21875-034, GIBCO) supplemented with 10% FBS onto a 50 mL falcon tube through a 70-μm cell strainer. Cells were centrifuged at 300×*g* for 5 min. The supernatant was discarded and cells were then incubated for 2 min with an Erythrocyte Lysis Solution (150 mM NH_4_Cl, 1 mM KHCO_3_, 0.1 mM EDTA). After washing with cold PBS supplemented with 2% FBS, cells were incubated at 4 °C for 15 min with a mix of FcBlock (1/200; Clone 2.4G2; BD Pharmingen; BD Bioscience), and the antibody Ly6G (clone 1A8, FITC; BD Pharmingen) with 10 µL/10^7^ cells. Cells were washed with PBS-0.5% BSA, and were then incubated with anti-FITC MicroBeads (#130-048-701, Miltenyi Biotec) for 15 min at 4 °C with 10 µL microbeads/10^7^ cells. After washing, the fraction of positive Ly6G cells was magnetically collected and prepared for immediate use or cells were frozen in FBS serum with 10% of DMSO until the day of the experiment. Human neutrophils were isolated from the blood by density gradient centrifugation. Human and mouse neutrophils were stained with CellTracker™ Green CMFDA (#C2925; ThermoFisher Scientific).

### Time-lapse microscopy studies

Isolated and stained neutrophils (75,000 cells/mL) were added to the adult microglia cultures at 7DIV. Automated multiposition live cell imaging was carried out using a Leica TCS SP5 confocal microscope (Leica Microsystems, Heidelberg, Germany) equipped with Adaptive Focus Control to keep the specimen in focus and an incubation system with temperature and CO_2_ control. Cells were subjected to a time-lapse study while maintained at 37 °C in a humidified atmosphere of 5% CO_2_. All images (3–4 z sections) were acquired using a APO 63 × (numerical aperture 1.3) glycerol immersion objective lens, pinhole set at 1.5 Airy units. Images of CMFDA and DsRed were acquired sequentially line by line using 488 and 561 laser lines and detection ranges at 500–550 and 570–650, respectively. Simultaneously, bright field images were acquired. Multiposition confocal images were acquired every 4 min during 12–14 h, with an image matrix of 512 × 512 pixel; 600 Hz; 2 × line average and autofocus control. Manual analysis was performed using FIJI software (Version 2.0.0-rc-67/1.52d). We recorded 3–4 time-lapse videos per well and analysed 180–210 frames in each video. In every frame, manual tracking of neutrophils was performed using the MTrackJ plugin [45] to identify phagocytosis of neutrophils by microglial cells. We studied in parallel four wells per genotype (CSF1R^+/+^ or CSF1R^+/−^ microglial cells) in each independent experiment and conducted five independent experiments. The analysis was performed in a blinded fashion by assigning a code to each video that did not reveal the identity of the genotype.

### Phagocytosis assay with fluorescent beads

We used green fluorescent zymosan A bioparticles (#Z-23373; Thermo Fisher Scientific) in the phagocytosis assay. At 7 DIV, microglial cells were exposed to zymosan fluorescent beads (75,000 particles/mL) for 1 h. Following 3–4 washes to remove all the non-phagocyted particles, cells were fixed with cold 4% paraformaldehyde for 20 min, permeabilized with 0.2% Triton X-100 (Sigma) in PBS 0.1 M for 15 min, blocked with 3% goat serum in PBS for 1 h, and incubated overnight at 4 °C with the primary rabbit antibody against the P2RY12 receptor (1:200, #AS55042A, Anaspec). The next day, cells were washed and incubated with red fluorescence Alexa Fluor^®^ 546 dye-labelled goat anti-rabbit IgG antibody (#A10036, Life Technologies) for 1 h at room temperature. DAPI (#D3571, Life Technologies) stained was performed to visualize the cell nuclei. Cells were then covered using Fluoromount-G^®^ (Southern Biotech, Birmingham, AL, USA). Images were obtained with a fluorescence inverted microscope (Leica CTR 40000).

### Immunofluorescence in brain tissue sections

Mice were perfused via the left heart ventricle with 40 mL of cold saline (0.9%) followed by 20 mL of cold 4% paraformaldehyde (PFA) diluted in phosphate buffer (PB) pH 7.4. The brain was removed, fixed overnight with the same fixative, and immersed in 30% sucrose in PB for cryoprotection for at least 48 h until the brains were completely sunk to the bottom of the tube. After that, brains were frozen in isopentane at − 40 °C. Cryostat brain sections (14-μm thick) were fixed in ethanol 70%, blocked with 3% normal serum, and incubated overnight at 4 °C with primary antibodies: rat monoclonal antibodies against Ly6G (clone 1A8, 1:100, #127601, Biolegend) or NIMP-R14 (anti-Ly6G/C, 1:100, #ab2557, Abcam); goat polyclonal antibodies against α4-laminin (1:50, #AF3837, R&D), or PDGFRβ (1:100, #AF1042; R&D); rabbit polyclonal antibodies against P2RY12 (1:250, #AS-55043A, AnaSpec Inc.), ionized calcium-binding adapter molecule-1 (Iba-1) (1:100, #016-20001, Wako Chemicals), glial fibrillary acidic protein (GFAP) (1:400, #Z0334, Dako), or pan-laminin (1:100, #Z0097, Dako). To amplify the signal of the DsRed cells we used a goat polyclonal anti-DsRed antibody (#sc-33354, Santa Cruz Biotechnology, Inc.) diluted 1:100. The secondary antibodies were: Alexa Fluor 488, 546, or 647 (Molecular Probes; Life Technologies S.A.) diluted 1:500. Cell nuclei were stained with DAPI or To-Pro3 (Invitrogen). Cryostat sections from human brain tissue were processed for immunofluorescence as described above with a rabbit polyclonal antibody against P2RY12 (1:200, #5042A, AnaSpec) and a mouse monoclonal antibody against Ki67 (1:400, #9449, Cell Signaling Tech). Consecutive sections were stained with thionine for examination of the lesion at the light microscope. Confocal images were obtained (TCS-SPE-II or SP5 microscopes from Leica Microsystems; or a Zeiss LSM880 microscope) and were not further processed except for enhancing global signal intensity in the entire images for image presentation purposes using LAS software (Leica), ImageJ, or Adobe Photoshop. For estimation of the density of P2RY12^+^ cells and Ki67^+^ cells in human brain sections, images were obtained (40 × objective), the number of immunostained cells and cell nuclei per image were counted in ten different fields per brain region of each subject, and average values per region and time group were calculated. For cell counting in mouse brain sections, we obtained 5–6 confocal images of the immunostaining (63 × objective) in three different brain sections per mouse.

### Analysis of microglia morphology

Microglia morphology was assessed using FIJI software (Version 2.0.0-rc-67/1.52d) and IMARIS software (IMARIS BITPLANE v.9.0). Basic shape descriptors such as the Circularity Index (CI) or the area were performed with the plugin Shape Descriptors [68]; other parameters, such us the Ramification Index (RI), were obtained using the Sholl analysis plugin [21]. Parameters such as volume or sphericity index were measured using Imaris Software after creating a 3D surface in the maximum intensity projection image. Then, microglial cells were thresholded by the Huang method [34] to generate a binary mask (with a 1.5 mean filter). The CI parameter was calculated by the Shape Descriptors plugin (4p[area]/[perimeter]^2^). The highest count of intersections (Max inters) reflects the highest number of processes in the cell.

### Statistics

Two-group comparisons were carried out with the Mann–Whitney *U* test. For multiple group comparisons we used the Kruskal–Wallis test followed by the Dunn’s test. Comparisons were two-sided. Comparisons of groups by brain region and time were carried out with two-way ANOVA followed by the Bonferroni post-hoc analysis. Two-way ANOVA by genotype and experiment, with an experiment-matched design, was used to analyze quantification of in vitro studies. Statistical analyses were performed with GraphPad software. The specific test used in each experiment and n values are reported in the figure legends.

## Results

### Microglia cells degenerate in the core of infarction

**Fig. 1:**
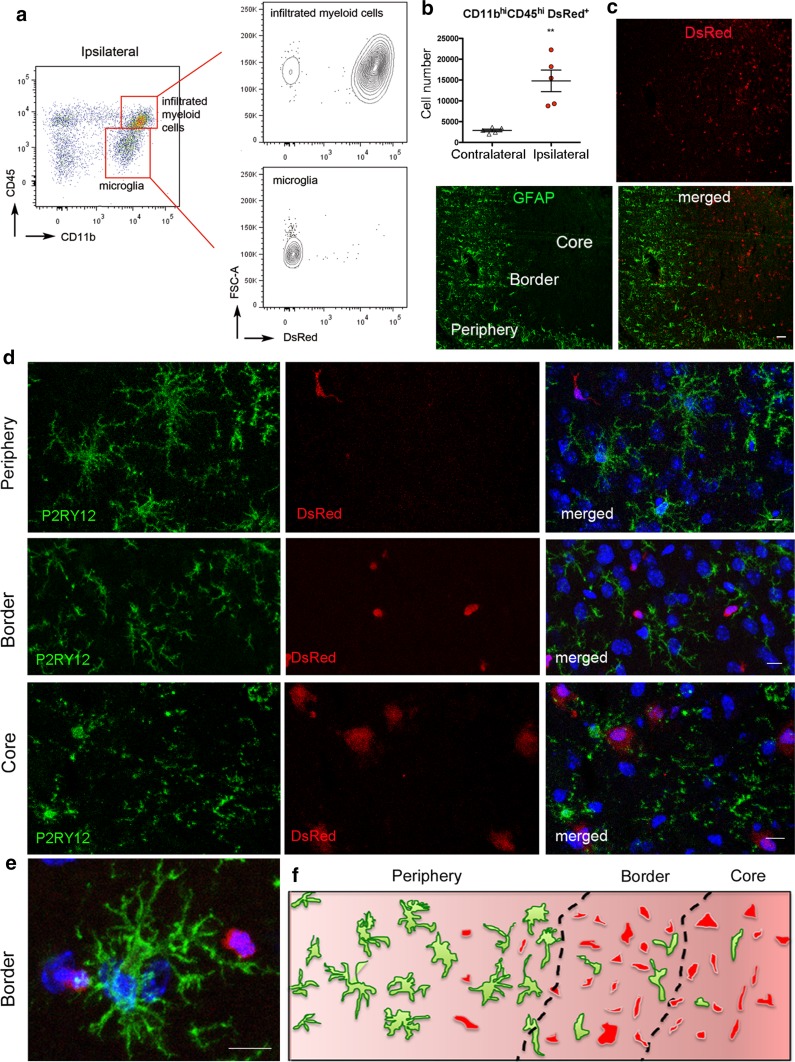
Localization of microglia and infiltrating leukocytes. We generated chimeric mice by administering DsRed fluorescent bone marrow cells to 2-month old wild-type receptor mice (*n* = 10). After 2 months, we induced ischemia and 4 days later we studied the brain by immunofluorescence (*n* = 5) (**a**, **b**) and flow cytometry (*n* = 5) (**c**, **e**). **a** CD45^hi^CD11b^hi^ cells infiltrating the ipsilateral hemisphere are mostly DsRed^+^ whereas CD45^dim^CD11b^dim^ microglial cells are DsRed^−^. **b** Flow cytometry shows an increase in infiltrating DsRed leukocytes (CD11b^hi^CD45^hi^) (Mann–Whitney test, **p = 0.008). **c** Immunostaining of astrocytes (GFAP, green) showed the presence of DsRed cells at the border and core of the lesion separated from the peripheral area that shows a prominent astroglial reaction. **d** Microglial cells were stained with an antibody against P2RY12 (green), which did not co-localize with DsRed^+^ leukocytes. The morphology of the microglia in the different regions is illustrated with representative images. Cell nuclei were stained with To-Pro3 (blue). **e** P2RY12^+^ microglial cell at the border of the lesion nearby DsRed^+^ infiltrating leukocytes. **f** Schematic representation of the distribution of microglia (green) and infiltrating leukocytes (red) in the different regions of the ischemic hemisphere. Scale bar **c** 50 µm; **d**, **e** 10 µm

To unequivocally distinguish microglia from infiltrating leukocytes we generated chimeric mice with the hematopoietic system derived from fluorescent (DsRed) donor mice where microglia remained non-fluorescent, whereas a high proportion of peripheral myeloid cells were DsRed^+^ (Fig. 1a; Online Resource 2). Most myeloid cells infiltrating the ipsilateral brain hemisphere were DsRed^+^ 4 days after MCAo, as assessed by flow cytometry (Fig. 1a, b), and they were preferentially located in the lesion core where the expression of GFAP is lost (Fig. 1c). We stained microglia with anti-P2RY12 antibodies [7, 28, 59] and verified that the infiltrating DsRed^+^ cells were not P2RY12^+^. Microglia and infiltrating DsRed^+^ leukocytes co-existed at the border of infarction, whereas microglia were abundant at the infarct periphery but scarce in the core of the lesion (Fig. 1d–f). Microglia acquired a reactive phenotype at the periphery and border of infarction with thicker ramifications compared to microglia of the contralateral hemisphere. In contrast, microglial cells in the infarcted core showed a dystrophic morphology since the cell body became smaller and there were only a few long ramifications showing an appearance of discontinuity as if they were broken or beaded, and the density appeared to be reduced (Fig. 1d).

**Fig. 2:**
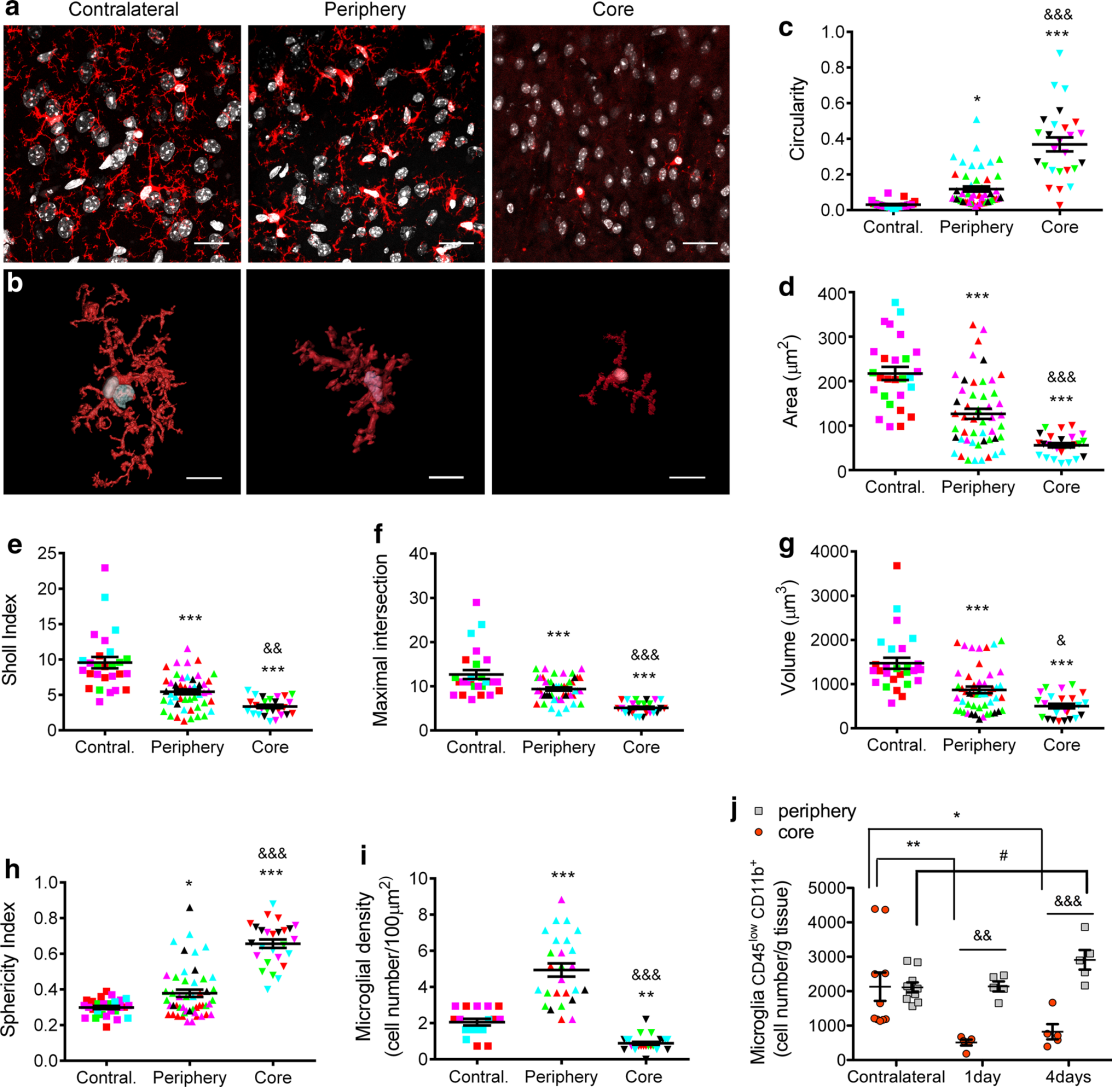
Morphology and number of microglial cells in the different brain regions after MCAo. **a**, **b** Images of microglia (red) of CX3CR1^cre/ERT2^-Rosa26:tdT mice (*n* = 3) in different brain regions. Nuclei are stained with DAPI (white). Confocal microscope images (**a**) and 3D-reconstruction (Imaris) of a representative microglial cell per region (**b**). **c**–**f** Analysis of microglial morphology in the core and periphery of the ipsilateral cortex and the contralateral cortex (Contral.) (*n* = 25–49 cells per region of 5 different mice represented by the different colors) using ImageJ tools. **g**, **h** Imaris analysis of 3D-reconstructions of the above cells (**b**). **i** Counting the number of microglial cells per area showed an increased microglial density in the periphery and a decrease in the core (*n* = 17–27 fields in 5 different mice). Statistical analyses in **c**–**i** were carried out with the Kruskal–Wallis test followed by the Dunn’s test. **p < 0.01 and ***p < 0.001 vs. contralateral microglia; ^&&^*p* < 0.01 and ^&&&^*p* < 0.001 vs. peripheral microglia. **j** Flow cytometry after dissecting out the core and periphery regions of the ipsilateral hemisphere and mirror regions of the contralateral hemisphere 1 and 4 days after MCAo in an independent group of mice (*n* = 5 mice per time point). For comparative purposes the number of microglial cells (CD45^low^CD11b^+^) is expressed by mg of brain tissue. Data analysis was conducted with two-way ANOVA by region (*p* < 0.0001) and time point (*p* = 0.039) followed by the Bonferroni test. The number of microglia was higher in the periphery than the core of infarction 1 day (^&&^*p* < 0.01) and 4 days (^&&&^*p* < 0.001) after MCAo. Furthermore, the number of microglia decreased in the core of infarction versus the corresponding contralateral region 1 day (**p < 0.01) and 4 days (*p < 0.05) post-ischemia. In the periphery, the number of microglia cells increased in the ipsilateral versus the corresponding contralateral hemisphere at day 4 (^#^*p* < 0.05). Scale bar 10 μm

To ensure that in wild-type mice we did not miss microglial cells in the infarcted core due to downregulation of the markers used to label microglia, i.e. Iba1 and P2RY12, we studied the CX3CR1^cre/ERT2^ mice [74] crossed with floxed Rosa26:tdT reporter mice [42], which express the red fluorescent protein in microglia (Fig. 2a, b; Online Resource 3) obtaining the same findings as in wild-type mice. Next, we analysed microglia morphology (Online Resource 4) in the contralateral hemisphere, and in different zones of the ipsilateral hemisphere, i.e. periphery and infarcted core, 4 days after MCAo (Fig. 2c–h). Shape descriptors showed increased circularity and reduced area of microglia in the ipsilateral hemisphere that was more marked in cells located in the core region (Fig. 2c, d). A Sholl analysis showed that ischemia reduced the number of ramifications, and maximal intersections per microglial cell, and again the changes were greater in the core (Fig. 2e, f). Analysis of 3D-reconstructions of the cells showed a reduced volume and higher sphericity index after ischemia, particularly in the core (Fig. 2g, h). Furthermore, the quantification of cells per area showed that while microglial cell density increased in the periphery, it was reduced in the core of the lesion (Fig. 2i). This result was confirmed by flow cytometry after excising the core and periphery of the ipsilateral hemisphere and mirror regions of the contralateral hemisphere 1 and 4 days after MCAo. Microglia cell number was severely reduced in the core of the lesion at 1 and 4 days post-ischemia (Fig. 2j). Altogether, these findings show that microglial cells are sensitive to persistent ischemic conditions and are lost in the infarcted core.

### Microglia cells proliferate at the periphery of infarction

**Fig. 3:**
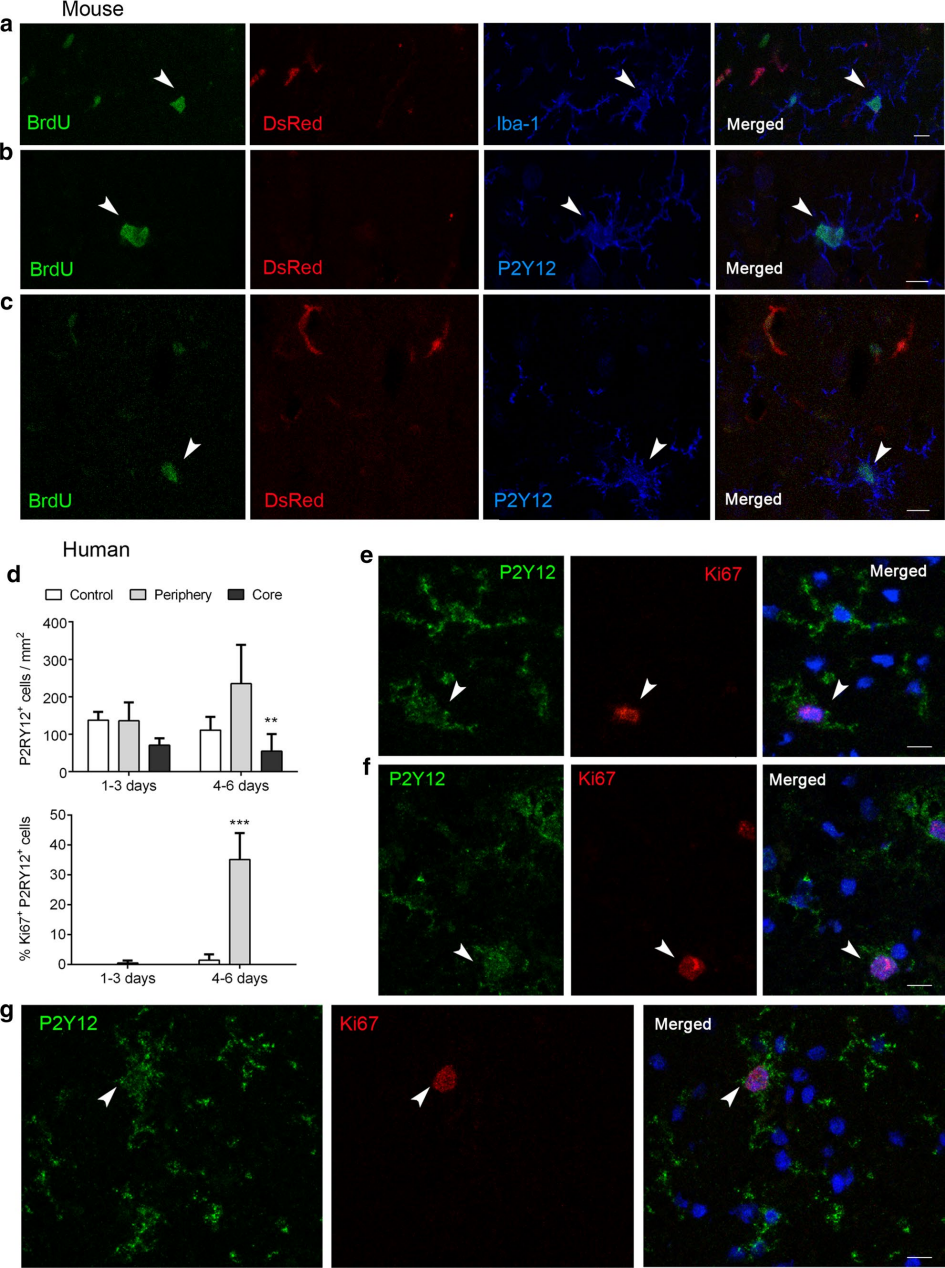
Microglial proliferation after ischemic stroke in mouse and human brain. Immunofluorescence in mouse (**a**–**c**) and human (**d**–**g**) brain tissue. **a**–**c** BrdU incorporation (green) in DsRed^−^ ramified microglial cells expressing P2RY12 (blue). **d** Human tissue of stroke patients deceased at different time points after stroke onset. The brains were grouped according to the day of death after stroke in ‘1–3 days’ (*n* = 3) and ‘4–6 days’ (*n* = 3) groups. The number of P2RY12^+^ cells per area tended to decrease in the core of the lesion in both groups and to increase at the periphery of infarction in the ‘4–6 days’ group. **p < 0.01 vs. periphery. The percentage of Ki67^+^ cells among the P2RY12^+^ cell population increased at the periphery of infarction in the ‘4–6 days’ group (***p < 0.001 vs. control and vs. core). **e**–**g** Representative images of double-immunopositive P2RY12 (green) and Ki67 (red) microglial cells (arrowheads) at the periphery of infarction from different stroke patients deceased 5 (**e**–**f**) or 6 (**g**) days after stroke. Nuclei are stained with To-Pro3 (blue). Scale bar 10 µm

In contrast to the lesion core, the microglial cell number increased at the periphery of infarction 4 days post-ischemia (Fig. 2i, j). This effect was attributable to microglial proliferation as shown in DsRed chimeric mice that received injections of the cell proliferation marker BrdU after MCAo (Fig. 3a–c). By cell counting, we calculated that 26.8 ± 8.8% (mean ± SD, *n* = 3 mice) of the microglial cells at the periphery of infarction incorporated BrdU suggesting microglial proliferation, in agreement with previous reports [14, 40]. In contrast, microglial cells were BrdU^−^ in the contralateral non-ischemic hemisphere. Notably, BrdU was found in ramified microglial cells suggesting that microglia could undergo cell division without regression of their differentiation status. We extended the results to the human brain by showing an increased number of microglial cells and proliferating Ki67^+^ microglia at the periphery of infarction in post-mortem human brain tissue of stroke patients (Fig. 3d–g). We also found reduced microglial cell numbers in the lesion core of stroked human brains (Fig. 3d) thus supporting that the findings in mice might be relevant to human stroke.

### Reactive microglial cells engulf infiltrating neutrophils at the periphery of infarction

**Fig. 4:**
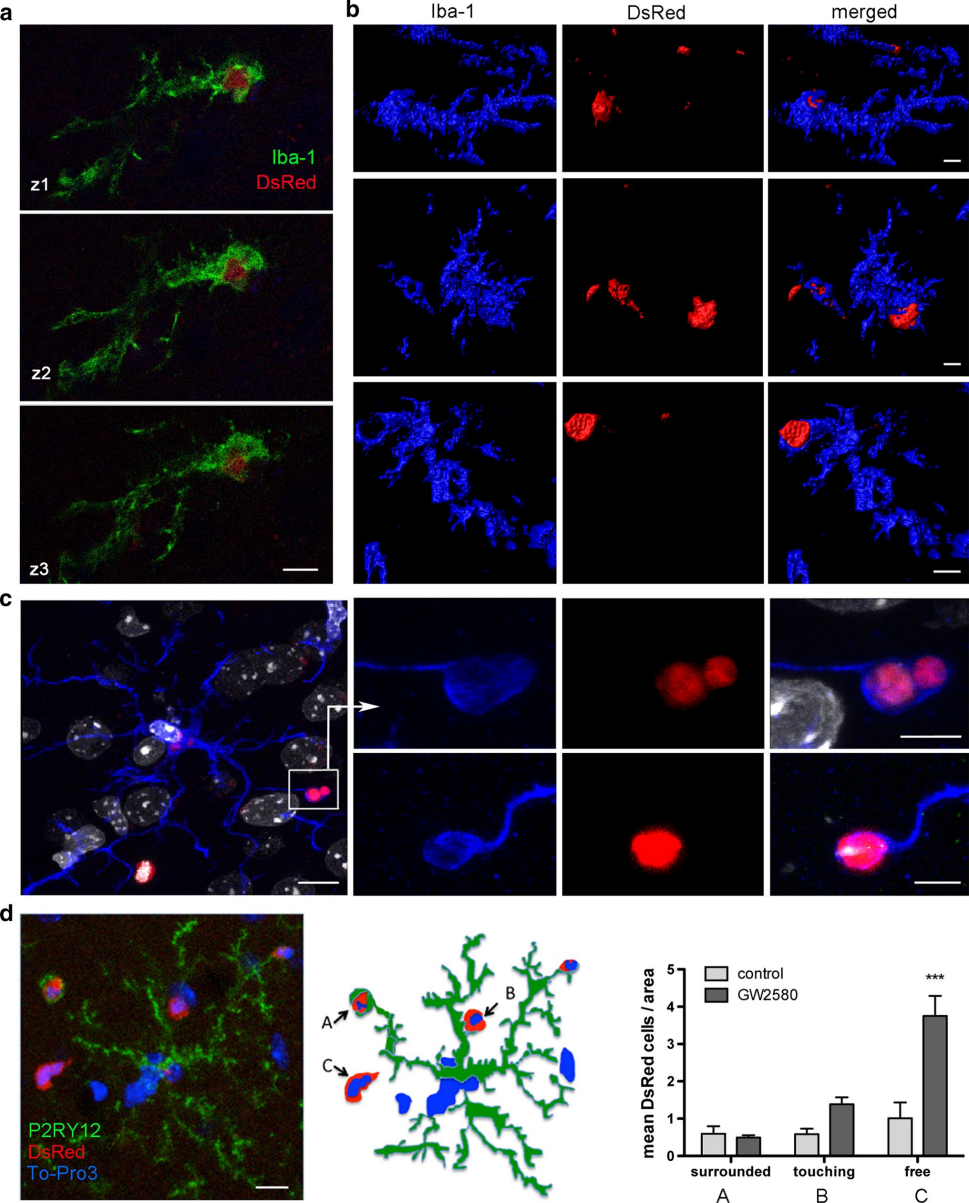
Microglia cells phagocytose infiltrating DsRed leukocytes. **a**–**c** Chimeric mice were generated by bone marrow transfer from donor DsRed mice to recipient wild type mice and were subjected to ischemia (*n* = 5). Immunofluorescence showing Iba1^+^ DsRed^−^ microglial cells engulfing DsRed leukocytes at the periphery of infarction at day 4 after MCAo. **a** Confocal images of an Iba1^+^ (green) DsRed^−^ microglial cell engulfing a DsRed leukocyte. The images correspond to different *z* planes. **b** 3D-reconstructions of Iba1^+^ DsRed^−^ microglial cells (blue) engulfing DsRed^+^ leukocytes (red). **c** Microglial cell (Iba1^+^, blue) with engulfed red cells in several prolongations. Nuclei are stained with DAPI (white). Magnified details of engulfed DsRed cells are shown on the right hand side. **f** DsRed chimeric mice received oral administration of either the CSF1R inhibitor GW2580 or the vehicle (*n* = 5 per group) starting 2 h prior to MCAo and then daily for 3 days. The brain was studied 4 days post-ischemia by immunofluorescence using anti-P2RY12 antibody to label microglia (green), DsRed for infiltrating leukocytes, and To-Pro-3 for staining the cell nuclei (blue). The image shows a microglial cell at the periphery of infarction engulfing DsRed leukocytes. Schematic representation of this cell illustrating: (A) a DsRed cell completely surrounded by a microglial process; (B) a microglial process making apparent contact with a DsRed leukocyte (touching); and (C) a Dsred leukocyte separated from microglia (free). Counting the number of DsRed cells in the above status (A-C) in relation to microglia showed that the CSF1R inhibitor increased (***p < 0.001) the number of free DsRed cells. Two-way ANOVA by treatment and condition followed by the Bonferroni test. Scale bar corresponds to **a**, **b** 5 µm; **c** 10 µm for left image and 5 µm for the magnifications on the right; **d** 10 µm

Our data identified the edge of the lesion as a site for possible interactions between microglia and leukocytes since both cell types were abundant in this zone. Indeed, we observed numerous microglial cell processes surrounding the blood vessels and getting into contact with infiltrating DsRed^+^ leukocytes by sampling the DsRed^+^ cells adjacent to the vascular endothelium (Online Resource 5). Furthermore, we detected microglial processes surrounding DsRed^+^ cells suggestive of engulfment of leukocytes (Fig. 4a–c; Online Resource 6). 3D-reconstructions of confocal images showed phagosomes completely wrapping DsRed^+^ leukocytes (Fig. 4b, c). The CSF1R signaling pathway is critical for survival of microglia and maintenance of their functions [2], including endocytic processes [60]. Consequently, by interfering with microglial function after oral administration of the CSF1R inhibitor GW2580 [12] for 4 days, we found more DsRed^+^ cells at the periphery of infarction not surrounded by microglia or in apparent contact with microglia, suggesting that microglia dysfunction impaired the process of phagocytosis of leukocytes (Fig. 4d).

**Fig. 5:**
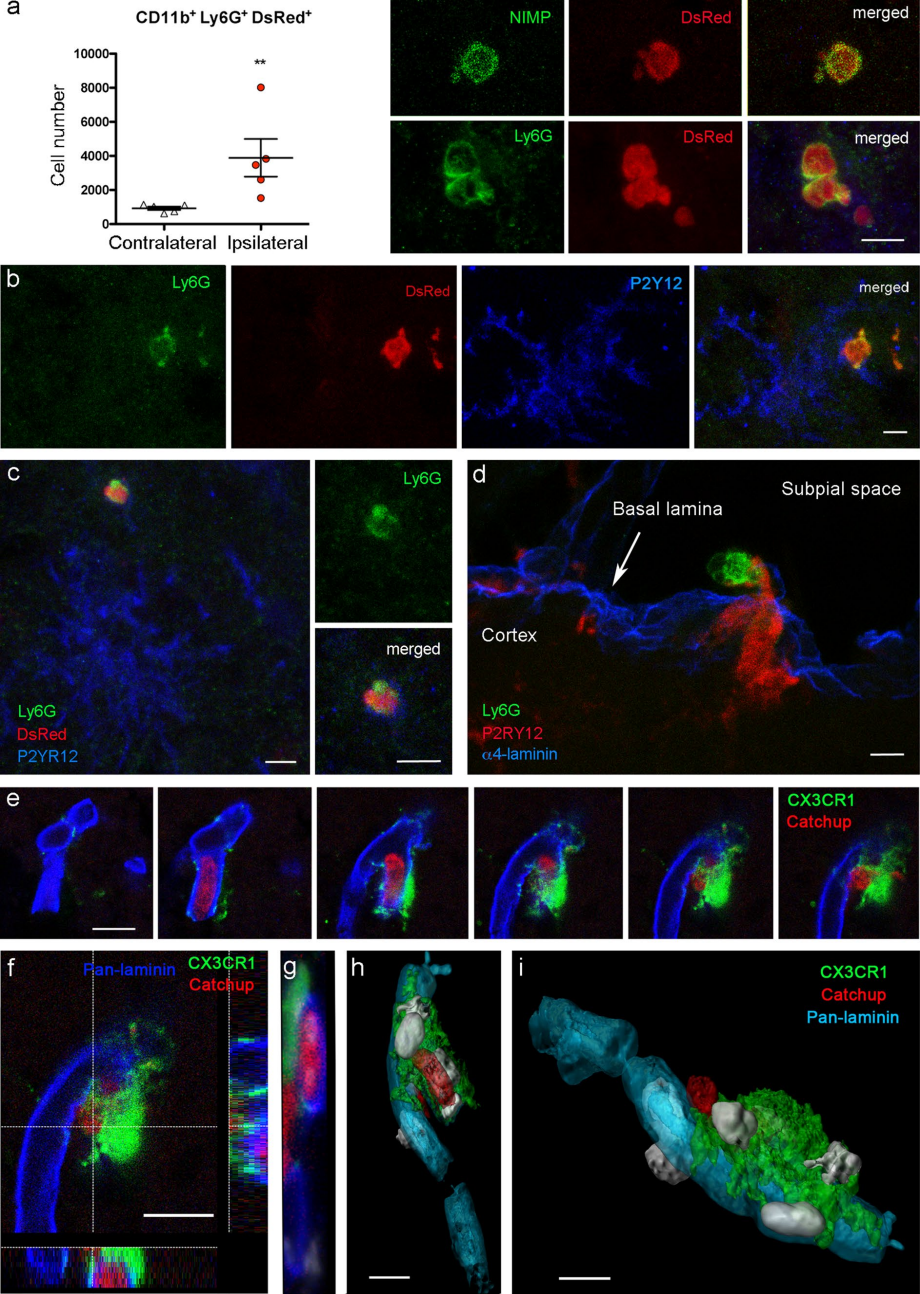
Microglia engulf neutrophils. **a**–**c** Chimeric mice generated by transfer of DsRed bone marrow cells to wild type recipients. **a** Flow cytometry shows CD11b^+^ Ly6G^+^ DsRed neutrophils in the ipsilateral but not the contralateral hemisphere (Mann–Whitney test, **p = 0.008, *n* = 5) 4 days post-ischemia. Infiltrating neutrophils (NIMP-R14^+^, Ly6G^+^) are DsRed^+^. **b**, **c** P2RY12^+^ microglial cells (blue) engulf neutrophils at the periphery of infarction. Images obtained 4 days after MCAo representative of *n* = 5 chimeric mice. **d** Image of a CX3CR1^cre/ert2^:Rosa26-tdT mouse 4 days after MCAo showing a microglial cell (red) sending a process across the external basal lamina of the cortex (α4-laminin, blue) to trap a neutrophil (Ly6G^+^, green) in the subpial space. **e**–**i** Images obtained from CX3CR1^gfp/+^/Catchup mice, which have gfp^+^ microglia (green) and tdT^+^ neutrophils (red) 1 day after MCAo. Sequence of confocal z-images showing the basal lamina (pan-laminin, blue) of a capillary with a neutrophil in the lumen and an extravasated neutrophil, surrounded by a microglial cell (**e**). *z* projections of one plane and confocal projection illustrating interaction of microglia with basal lamina and neutrophils (**f**–**g**). 3D-reconstructions showing the microglial cell (green) attached to the capillary (blue) and intraluminal (**h**) and extravasated (**i**) neutrophils (red). Nuclei are stained with DAPI (white). Images were obtained from superficial cortical layers (**a**–**c**, **e**–**i**) and the brain surface (**d**) in zones corresponding to the core of the lesion (**a**), border of the lesion (**d**–**i**), and periphery (**b**, **c**). Scale bar 10 μm

Neutrophils showed bright DsRed fluorescence in the DsRed chimeric mice, as assessed by flow cytometry and immunofluorescence 4 days post-ischemia (Fig. 5a). We detected Ly6G^+^ DsRed^+^ neutrophils shrouded by microglia at the periphery of infarction (Fig. 5b, c). Furthermore, processes of microglial cells located nearby the surface of the cortex seemed to traverse the external cortical basement membrane and surround neutrophils located in the subpial space (Fig. 5d). Catchup^IVM^ mice crossed with transgenic Cx3Cr1^gfp/gfp^ mice allowed obtaining heterozygous double reporter mice with red fluorescent neutrophils and green fluorescent microglia [50, 75]. In these mice, we observed microglia (green) adjacent to the basal lamina of blood vessels and surrounding extravasated neutrophils (red) after MCAo (Fig. 5e–i; Online Resource 7).

### Microglia phagocytose neutrophils in vitro and the process is impaired in CSF1R^+/−^ microglia

**Fig. 6:**
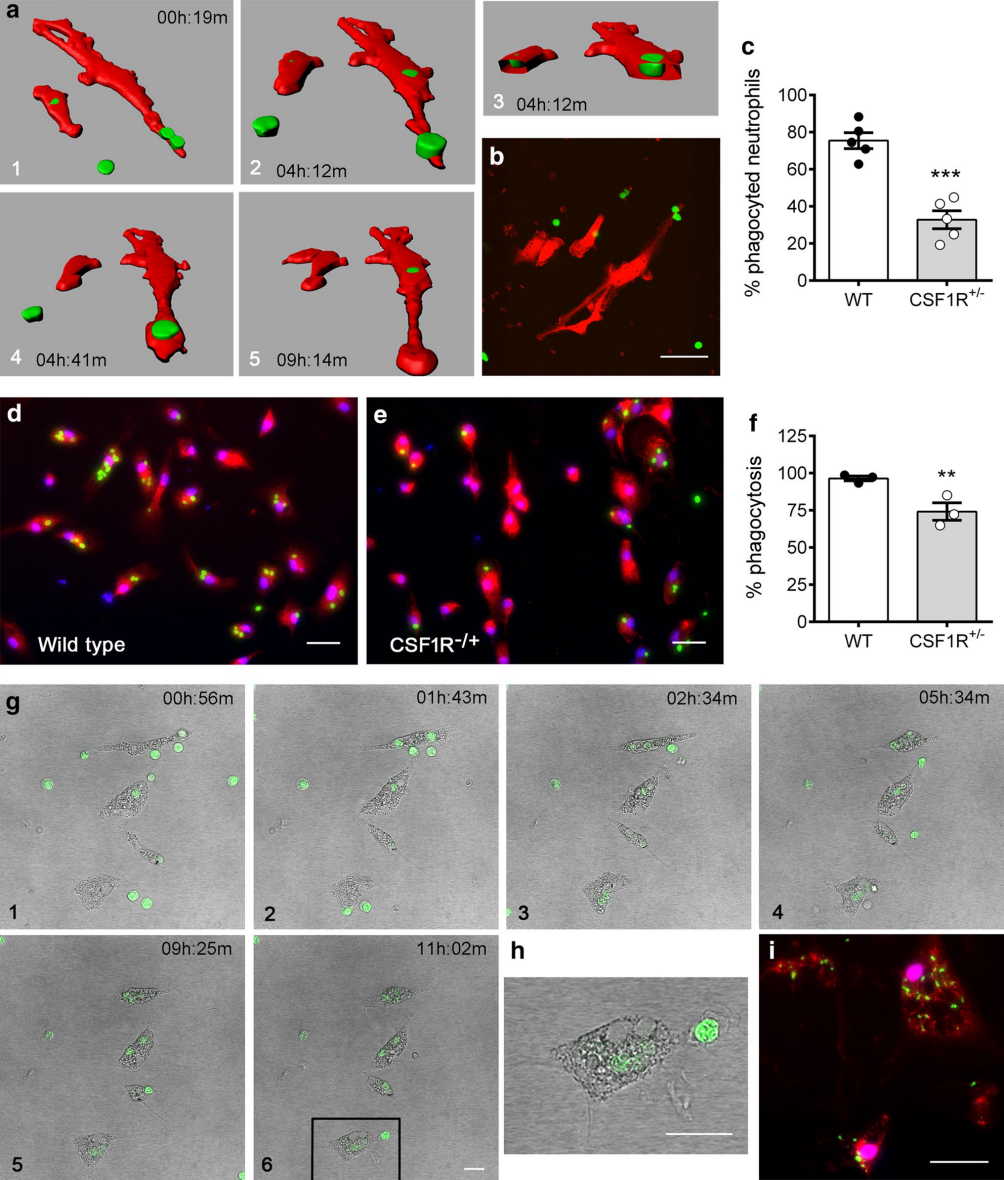
Microglia phagocytose neutrophils in vitro under the control of microglial CSF1R expression. Microglial cells were obtained from adult mice (**a**–**f**) or a human stroke patient deceased 5 days after ischemic stroke (**g**–**i**). After 7 days in culture, the cells were exposed to corresponding control mouse or human neutrophils and studied by time-lapse confocal microscopy for 14 h. **a**, **b** Microglia were obtained from DsRed mice (red) and neutrophils were stained with CMFDA (green). 3D-reconstructions of image sequences (1–5) of the time-lapse video (see supplementary video 3 and 4) illustrating the phagocytosis of neutrophils by mouse microglia (**a**). The time point of each image is indicated (hours:minutes). An original confocal image is shown **b** for illustrative purposes. **c** Microglia cultures were obtained from CSF1R^+/+^ (WT) and CSF1R^+/−^ littermate mice (*n* = 5 mice per genotype) and the cell cultures were exposed to neutrophils and studied by time-lapse microscopy, where microglial cells were seen by phase contrast and neutrophils were stained with CMFDA and detected by green fluorescence. We studied four wells per mouse in each independent experiment (*n* = 5), recorded 3–4 videos of different fields per well, and analysed 180–210 frames per time-lapse video. Quantification of the number of neutrophils phagocytosed by microglia (normalized by the number of microglia in each well) shows that heterozygous CSF1R^+/−^ microglia phagocytose less neutrophils than WT microglia. Two-way ANOVA by genotype and experiment, with an experiment-matched design, show a genotype effect ***p < 0.0001. **d**–**f** Cultures of WT and CSF1R^+/−^ microglia were exposed to green fluorescent zymosan beads (*n* = 3 independent experiments). At the end of the experiment cells were fixed and immunostained with anti-P2RY12 antibodies (red) and the number of cells containing fluorescent beads was counted. Compared to WT microglia (**d**), CSF1R^+/−^ microglia (**e**) also shows a reduced capacity to phagocytose zymosan beads (green) (**f**). Two-way ANOVA by genotype and experiment, as above, shows a significant genotype effect **p < 0.01. **g** Sequential snapshot images (1–6) of human microglia obtained during the 12 h time-lapse microscopy study in which 720 frames were studied. The images illustrate neutrophil phagocytosis by human microglia at the indicated times (hours:minutes). **h** Magnification of the indicated part of sequence 6 in **g** is shown to illustrate the engulfment of a neutrophil by a human microglial cell. **i** After the time lapse-experiment, human cells were fixed and stained with anti-P2RY12 antibodies (red). Nuclei are shown in blue (DAPI). The microglial cells contain material (green fluorescence) derived from digested neutrophils. Scale bar 20 μm

To further investigate the phagocytosis of neutrophils by microglia, we carried out an in vitro study using time-lapse confocal microscopy. We isolated microglia from adult mouse brains, cultured the cells in vitro for 7 days, and exposed them to freshly isolated bone marrow neutrophils (stained with CMFDA) to study phagocytosis. Microglial cells phagocytosed neutrophils in vitro (Fig. 6a, b, and time-lapse microscopy movie in Online Resource 8). By following neutrophil trajectories along time (time-lapse microscopy movie in Online Resource 9), we counted the number of neutrophils phagocytosed by microglia. We validated that allogenicity in the phagocytosis experimental design was not interfering with the assay by comparing phagocytosis of neutrophils obtained from different mice with the phagocytosis of neutrophils from the same mouse. We found no differences, ruling out prime effects of allogenicity in the phagocytosis of neutrophils by microglia (Online Resource 10). Given that we observed signs of impaired phagocytosis of neutrophils in vivo after ischemia in mice after short-term treatment with a CSF1R inhibitor (Fig. 4d), we investigated in vitro the role of CSF1R in this process. To this end, we obtained microglia from adult mice with heterozygous CSF1R^+/−^ microglia (obtained by crossing CX3CR1^cre/ERT2^ with CSF1R^flox/+^ mice). Less neutrophils were phagocytosed by CSF1R^+/−^ microglia compared to CSF1R^+/+^ microglia, as assessed by time-lapse microscopy where we counted the neutrophils phagocytosed by microglia during the 14-h duration of the experiment (Fig. 6c). In addition, CSF1R^+/−^ microglia showed reduced phagocytic activity in a phagocytosis assay with fluorescent beads (Fig. 6d–f). We then studied the potential relevance of these findings for human cells by obtaining human microglia from the post-mortem brain of an ischemic stroke patient deceased 5 days after stroke onset. After maintaining the human cells in culture for 7 days, we exposed the cells to neutrophils obtained from blood of a donor control subject. We observed very active phagocytosis of neutrophils (Fig. 6g, h and time-lapse microscopy movie in Online Resource 11). By immunofluorescence after the ex vivo assay, we detected P2RY12 expression in the cells and observed that they contained material from neutrophils (Fig. 6i).

### Post-ischemic microglial dystrophy/loss was associated with neutrophil accumulation

**Fig. 7:**
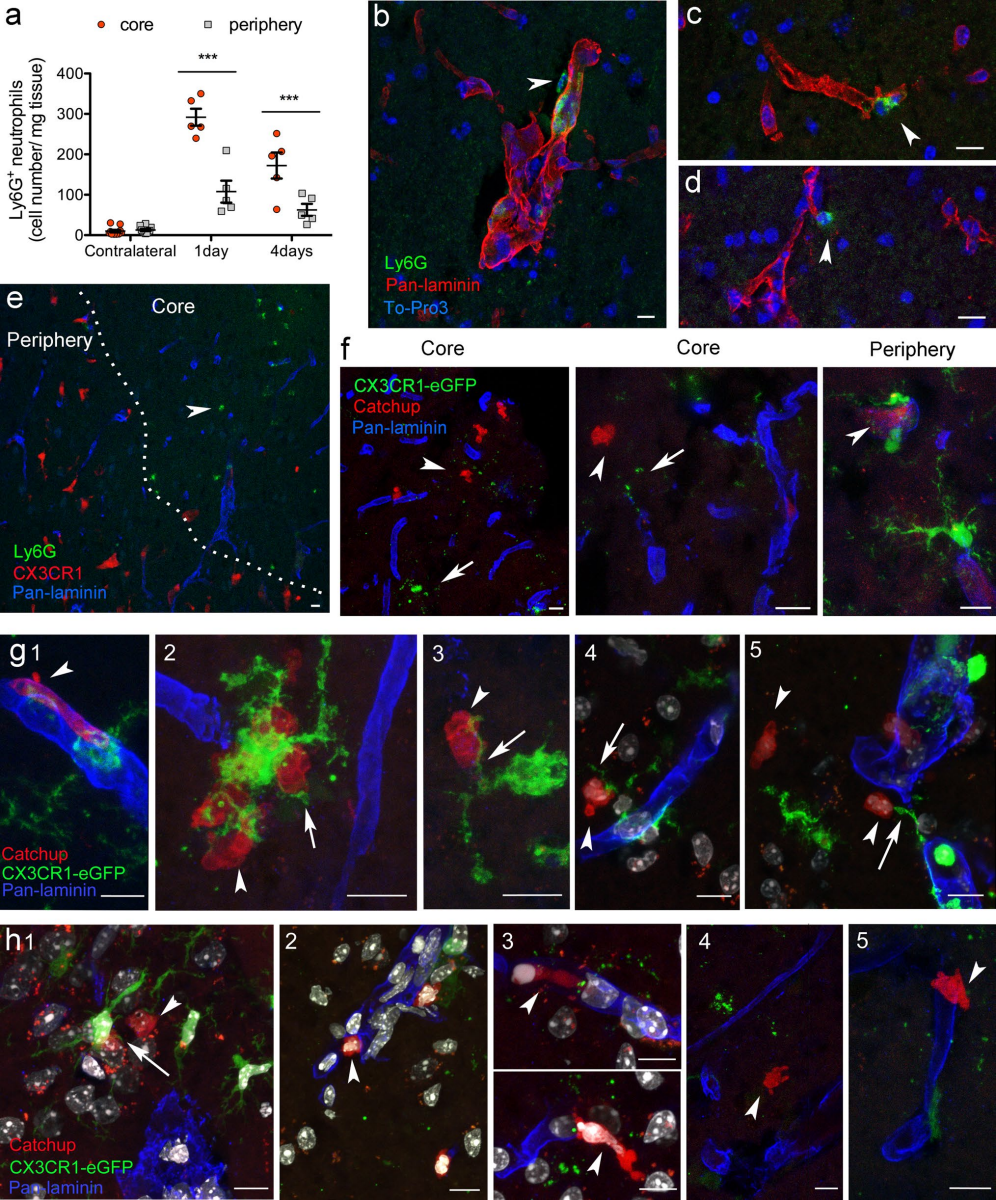
The presence of neutrophils in the brain parenchyma is associated with dystrophy and loss of microglia. **a** Flow cytometry analysis of CD11b^+^ Ly6G^+^ neutrophils in the core and periphery of infarction and in mirror regions of the contralateral hemisphere 1 and 4 days after MCAo (*n* = 5 mice per time point) shows that ischemia increases the number of neutrophils in the core of infarction more than the periphery (two-way ANOVA, Bonferroni test, ***p < 0.001). **b**–**d** Brain confocal images 1 day after MCAo (*n* = 8) show Ly6G^+^ neutrophils (green) in the perivascular space of a venule (**b**) and the parenchymal side of capillaries (**c**, **d**). The basal lamina is stained with Pan-laminin (red) and nuclei are stained with To-Pro3 (blue). **e** Neutrophil (Ly6G^+^, green) accumulation is higher at the border (dotted line) of the infarcted core than the periphery, whereas microglia (red cells, CX3CR1^cre/ert2^:Rosa26-tdT mice) accumulate in the infarct periphery and are scarce in the core. The vascular basal lamina is shown in blue (pan-laminin). **f**–**h** Images obtained from Catchup mice crossed with CX3CR1^gfp/gfp^ mice at day 1 (**f**, **g**) and day 4 (**h**) post-ischemia. Extravasated neutrophils (red) away from the vascular basal lamina (pan-laminin, blue) are seen in the core of the lesion where microglia (green) is absent or dystrophic, whereas neutrophils are surrounded by reactive microglial cells in the periphery (**f**). **g** Examples (1–5) of microglial cells sending prolongations towards neutrophils 1 day after pMCAo. Neutrophils located in perivascular spaces extend protuberances crossing the basal lamina (1). Extravasated neutrophils are seen near vessels with discontinuous basal lamina (2). They are surrounded by reactive microglia at the border of the lesion (2–4), but dystrophic microglia seem to be unable to fully reach the extravasated neutrophils (5). **h** At day 4, microglial cells sending prolongations towards neutrophils are seen at the border of the lesion (1). Extravasated neutrophils are seen in zones where microglia is dystrophic (1–2) or absent (3–5). The vascular basal lamina is hardly detected at places where the neutrophils are located (3). Arrowheads indicate neutrophils, whereas arrows indicate microglia. The cell nuclei (DAPI, white) are shown in **g** (4–5) and **h** (1–3). Scale bar 10 μm

Neutrophil numbers were higher in the core of infarction than the periphery 1 and 4 days after MCAo, as assessed by flow cytometry after dissection of these brain regions (Fig. 7a). Neutrophils were seen in perivascular spaces of venules (Fig. 7b) but also in the parenchyma outside the basement membrane in the core of the lesion (Fig. 7c, d). Strikingly, neutrophils were found in the brain parenchyma in zones of the core where microglia was absent or showed signs of dystrophy, as assessed in immunostained brain sections of wild-type mice and reporter mice, including the CX3CR1^cre/ERT2^-Rosa26:tdT mice (Fig. 7e) and the Catchup^IVM^-CX3CR1^+/−^ double reporter mice (Fig. 7f–h). At the periphery of infarction neutrophils were surrounded by reactive microglia, suggesting that microglia phagocytosed neutrophils thereby preventing their accumulation in this region. In contrast, in the core of infarction, dysfunction or loss of microglia might facilitate the accumulation of neutrophils.

### Microglia depletion increases the numbers of neutrophils in the ischemic brain tissue and augments brain injury

**Fig. 8:**
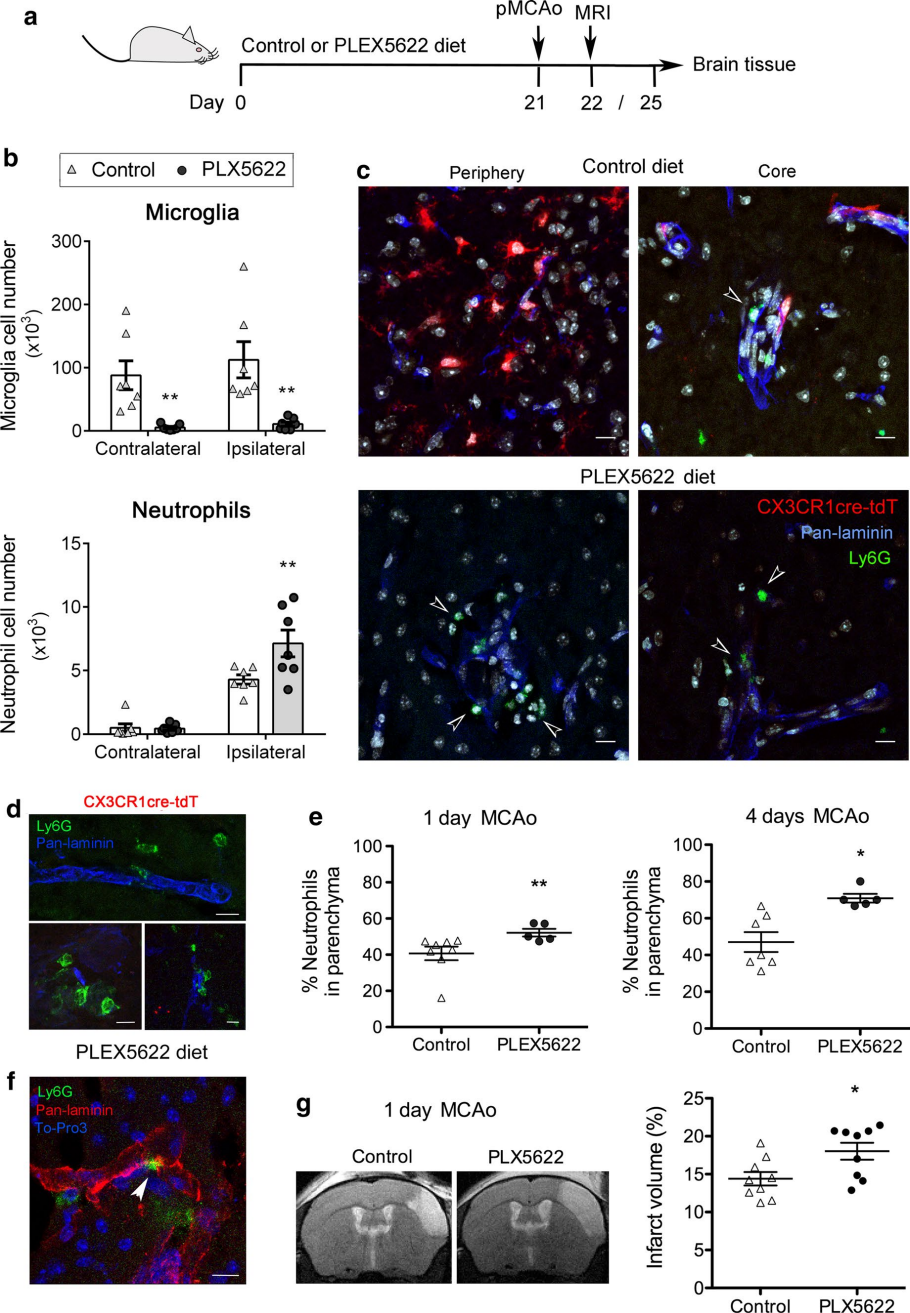
Microglia depletion increases the number of neutrophils in the brain parenchyma. **a** Schematic representation of the experimental design. Eight-week old mice received a control or PLX5622-containing diet for 21 days. Then MCAo was induced and the animals continued with the corresponding diet one or four more days until the end of the study. **b** Flow cytometry (*n* = 7 mice per group) showed the microglia (CD45^low^ CD11b^+^ cells) depleting effect of the PLX5622 diet, and an increased number of neutrophils (CD11b^+^Ly6G^+^) in the ipsilateral hemisphere of mice with depleted microglia. Two-way ANOVA by treatment and brain hemisphere, and Bonferroni post-hoc test, **p < 0.01. **c** Control or PLX5622-containing diet was given to CX3CR1^cre/ert2^:Rosa26-tdT mice with the same dosing regimen. Microglia is shown in red, neutrophils in green (Ly6G^+^), the vascular basal lamina is stained with Pan-laminin (blue), and nuclei (DAPI) are shown in white. The absence of microglia is associated with an increased presence of extravasated neutrophils (arrowheads). **d** Is a magnified image showing extravasated neutrophils after a PLX5622-containing diet. **e**, **f** Distribution of neutrophils in parenchymal versus perivascular or intravascular locations, as assessed by cell counting in brain sections immunostained with Ly6G, pan-laminin and To-Pro3. Mice treated with the PLX5622 diet show a higher (Mann–Whitney test) percentage of parenchymal neutrophils 1 day (**p = 0.004) and 4 days (**p = 0.006) post-ischemia (*n* = 5–7 mice per group). **f** Illustrates the previous staining after PLX5622 diet. **g** Brain lesion volume was measured with T2w MRI 24 h post-ischemia in both diet groups (*n* = 9 per group). The brain lesion was larger in mice depleted of microglia (Mann–Whitney test, *p = 0.031). Scale bar 20 μm (**c**); 10 μm (**e**, **f**)

To obtain further evidence that microglia remove neutrophils after ischemia, we depleted microglia by feeding the mice for 3 weeks with a diet containing a CSF1R antagonist (PLX5622) [15, 33, 69]. PLX5622 diet caused a strong reduction (90%) of the microglia population (Fig. 8a) but did not affect blood leukocyte counts (Online Resource 12 shows the gating strategy and cell quantifications are shown in Online Resource 13), in agreement with previous reports [20, 70]. However, we detected a reduction of a minor subset of blood Ly6C^−^ monocytes (Online Resource 13), which is dependent on CSF1R [39]. In brain tissue, PLX5622 diet reduced the numbers of infiltrating monocytes (40%) and F4/80^+^ macrophages (60%) versus the control diet 4 days post-ischemia (Online Resource 14), in agreement with previous findings suggesting a role for microglia in the recruitment of monocytes into the brain [20]. In contrast, the PLX5622 diet increased the numbers of neutrophils in the brain tissue after ischemia, as assessed by flow cytometry at day 4 (Fig. 8b). Immunofluorescence in CX3CR1^cre/ERT2^:R26-tdT mice showed extravasated neutrophils that seemed more abundant in the absence of microglia (Fig. 8c, d). By counting the number of neutrophils located either in the parenchyma or associated with blood vessels (hence not crossing the parenchymal basal lamina) we found an increase in the percentage of parenchymal neutrophils in the absence of microglia 1 and 4 days post-ischemia (Fig. 8e). Of note, neutrophils were often observed on the basement membrane of capillaries (Fig. 8d) and venules (Fig. 8f) suggesting a possible interaction of these cells with basal lamina components. In line with this, microglia depletion increased the size of the ischemic lesion (Fig. 8g), further supporting a beneficial effect of microglia at the periphery of the infarction.

## Discussion

This study supports the concept that microglia phagocytose and remove neutrophils after brain ischemia [14, 50–52] and demonstrates that neutrophil accumulation in the brain parenchyma is associated with reduced microglial phagocytic activity, attributable to ischemia-induced microglial cell dysfunction due to loss or dystrophy. Morphometric analysis of microglia showed changes in the periphery of the lesion compatible with microglial reactivity and similar to those reported [27]. Overall, morphological changes of microglia within the lesion core were larger than in the periphery, for instance regarding the notable loss of ramifications and reduced cell size. Such profound morphological changes of microglia in the lesion core might indicate a further process of transformation from reactive microglia to dystrophic microglia, potentially associated with cell dysfunction. Furthermore, we found reduced microglial cell numbers in the core of infarction. While our results support microglial degeneration in the infarcted core, microglial cells proliferated and accumulated at the periphery of infarction in mouse and human brain, in agreement with previous findings in the mouse brain [14, 40]. These reactive microglial cells at the periphery of infarction phagocytosed neutrophils, suggesting that the phagocytic activity of microglia prevented neutrophil accumulation in this region. Accordingly, the numbers of neutrophils were higher in the core than the periphery of the lesion. Microglial activity and survival are critically dependent on CSF1R [12]. Consequently, drug-induced inhibition of CSF1R or genetic reduction of CSF1R expression in microglia impaired their phagocytic capacity in vivo and in vitro. Previous studies reported that CSF-1 promotes phagocytosis of Ab1–42 peptide by primary human microglia in vitro [62], and it regulates cell motility in macrophages [58]. CSF1R is a tyrosine kinase that upon activation shows phosphorylation of several intracellular tyrosine residues [77]. Upon activation, CSF1R associates with several signaling molecules, notably phosphoinositide 3-kinase (PI3K) [58]. CSF1R also activates Akt [11], and it induces ERK1/2-mediated signaling in microglia [9]. Akt [22, 67] and ERK1/2 [19] are involved in the phagocytic process. However, the specific signaling molecules downstream of CSF1R participating in phagocytosis in microglia after brain ischemia, and the precise step(s) of the phagocytic process affected by CSFR1 remain to be identified. Microglia depletion induced by long-term inhibition of CSF1R in vivo [15, 33, 65, 69] increased the numbers of neutrophils in the ischemic brain tissue, further supporting the view that microglial cells contribute to neutrophil removal.

Neutrophils are attracted to the injured brain after acute stroke [10, 23, 30, 31, 50, 51]. Thereby, neutrophils adhere to venules and migrate through the vessel wall to reach perivascular spaces [17]. In addition, neutrophils access perivascular spaces of penetrating cortical vessels from the leptomeninges [55]. Accumulation of neutrophils in the leptomeninges might be due to extravasation from pial vessels. In addition, neutrophil migration from the skull bone marrow through direct anatomic connections [29] might explain the presence of neutrophils in the subarachnoid space, although migration of neutrophils from there to perivascular spaces of cortical vessels still needs further investigation. Subpial neutrophils are separated from the brain parenchyma by the basement membrane and glia limitans. Likewise, the parenchymal basal lamina and surrounding astrocyte end-feet separate perivascular cells from the brain parenchyma. Interestingly, we observed ramifications of microglial cells apparently crossing the basal lamina suggesting the possibility that reactive microglia might sample the perivascular space and also the subpial space after brain ischemia. Using intravital microscopy, we previously found evidence that microglia phagocytosed neutrophils before they extravasated to the brain parenchyma [50, 51]. However, further studies are required to demonstrate whether microglia can really cross the external cortical basement membrane after brain ischemia. Although we detected engulfment of complete cells by microglia, some of the images suggest that microglia may take portions of the neutrophils while they are located in the perivascular or subpial spaces, potentially through a process of trogocytosis [71].

The blood vessel glycocalyx and basement membrane composition varies between organs and inflammatory conditions suggesting that leukocytes may have to use diverse strategies to access different inflamed tissues [53]. In the brain, neutrophils cross the endothelial cell layer and the endothelial basal lamina of venules to reach the perivascular spaces after ischemia [17]. Then, they accumulate in the perivascular spaces because they do not seem to readily cross the parenchymal basal lamina [17], at least not at the same pace as they transmigrate through the former layers. However, the precise molecular determinants of this process remain to be identified. The different molecular composition of the two layers of basal lamina surrounding the perivascular spaces, local molecular diversity, and the finding that certain basal lamina components inhibit leukocyte transmigration [63], might explain why neutrophils have more difficulty to cross the parenchymal than the endothelial basal lamina after brain ischemia. In a model of transient ischemia, there is evidence suggesting that neutrophils are kept in the perivascular spaces without infiltrating the brain parenchyma [17], whereas other studies suggested that neutrophils reach the brain parenchyma [64]. It is plausible that stroke severity, status of microglia function, and time point of the study are critical determinants of the presence of neutrophils in the brain parenchyma. Neutrophils located in the perivascular spaces might damage the basement membrane by releasing proteolytic enzymes and/or undergoing NETosis [55]. However, at this stage we cannot exclude the possibility that neutrophils gained access to the brain parenchyma in a passive fashion after loss of vessel integrity in the ischemic core. Several lines of evidence support that after brain ischemia neutrophils release proteolytic enzymes, promote matrix metalloproteinase (MMP) activation, and cause BBB breakdown [25, 35, 36, 57, 66, 72]. Accordingly, blocking neutrophils or neutrophil-derived MMP-9 is markedly protective in models of systemic inflammation and stroke, e.g. [35, 44]. Furthermore, pharmacological inhibition of neutrophil elastase or genetic deficiency of this enzyme reduced BBB disruption and vasogenic edema after transient MCAo [64] suggesting that neutrophils contributed to vascular damage following stroke.

In this study we showed that, after permanent ischemia, neutrophils gained access to the brain parenchyma of the lesion core when it was already severely damaged and microglia was lost due to persistent ischemia. Under these conditions, parenchymal neutrophils might be bystanders of severe tissue damage. Therefore, it is likely that preventing the access of neutrophils to the brain parenchyma in this model would not have a major impact on the size of the brain lesion since the damage is already established by the time the cells reach the parenchyma and the core of infarction will not recover. This possibility agrees with the finding that inhibition or deficiency of neutrophil elastase was not protective in models of permanent MCAo [64]. In contrast, inhibiting microglial phagocytic activity in this model might bear negative effects by favoring neutrophil accumulation in the ischemic periphery. Accordingly, detrimental effects of neutrophils became apparent in our study after microglia depletion causing an abnormal increase in neutrophils and larger ischemic lesions. A limitation of our study is that we did not assess stroke outcome in the long term. Future work should investigate how microglia depletion affects the progression of the ischemic brain lesion and the neurological deficits. The results highlight an aspect of microglia phagocytic function that may be beneficial for the ischemic tissue. Nonetheless, several mechanisms can contribute to the detrimental effect of microglia depletion and CSF1R deficiency. For instance, pioneer studies demonstrated increased ischemic lesions related to reduced production of neurotrophic factors after depleting proliferating microglia [38], and neuroprotective functions mediated by CSF1R [47]. Moreover, we previously identified that absence of microglia significantly augmented infarct size in a model of transient ischemia, in part mediated by dysregulation of neuronal activity [65]. The latter model caused moderate leukocyte infiltration and we failed to observe a significant impact of microglia depletion on BBB injury and leukocyte recruitment, at least at the times examined [65]. In contrast to the findings suggesting beneficial effects of microglia in brain ischemia, several lines of evidence support that the phagocytic activity of microglia could exert negative effects by removing viable neurons through phagoptosis [5, 6, 48, 49]. It is possible that any negative consequences of phagoptosis of neurons might predominate under mild ischemic conditions where the inflammatory response is low, vascular integrity is preserved, and neutrophil attraction to the brain is negligible.

Collectively, our results support a model where microglia removes neutrophils from the parenchyma and perivascular and subpial spaces after brain ischemia. Severe ischemic conditions induce local microglia loss/dystrophy facilitating the presence of neutrophils in perivascular spaces first and in the brain parenchyma later. Overall, this study shows that reactive microglial cells phagocytose and remove neutrophils, whereas microglial loss or dysfunction enhances neutrophil accumulation in the ischemic lesion. Our results, hence, suggest that microglia function is critical to prevent neutrophil infiltration to the brain parenchyma and to minimize the negative impact of neutrophils in the vascular bed after ischemic stroke.

## Electronic supplementary material

Below is the link to the electronic supplementary material.

Online Resource 1. (Table) Clinical and radiological characteristics of the stroke patients. (PDF 84 kb)

Online Resource 2. (Figure) Flow cytometry analysis of blood and brain tissue of DsRed chimeric mice two month after bone marrow transfer of DsRed cells. (PDF 1258 kb)

Online Resource 3. (Figure) Flow cytometry analysis of tdT^+^ immune cells in CX3CR1^cre/ert2^ :Rosa26-tdT mice. (PDF 3076 kb)

Online Resource 4. (Figure) Morphological analysis of microglia. (PDF 341 kb)

Online Resource 5. (Figure) Microglia processes sample DsRed + cells located adjacent to the vascular endothelium. (PDF 333 kb)

Online Resource 6. (Movie) Imaris 3D reconstruction of a microglial cell (blue) containing phagocytosed DsRed^+^ cells. Nuclei are shown in white (DAPI). The image was obtained 4 days post-ischemia in a DsRed chimeric mouse. (AVI 18902 kb)

Online Resource 7. (Movie) Imaris 3D reconstruction of a microglial cell. Microglia (CX3CR1^+^, green) adjacent to the basal lamina of a capillary (pan-laminin, blue) with one intravascular and one extravasated neutrophil (Catchup^+^, red), in a double reporter mouse. The image was obtained 1 day after induction of ischemia. (AVI 5735 kb)

Online Resource 8. (Movie) Imaris 3D reconstruction of time-lapse confocal microscopy. The image shows phagocytosis of neutrophils by microglia. Microglia cells were obtained from adult DsRed mice (red cells). After 7 days in culture, fresh mouse bone marrow neutrophils were stained with CMFDA (green) and were added to the culture system. Total recorded time is 14 h. (AVI 6530 kb)

Online Resource 9. (Movie) Cell tracking. Example to illustrate neutrophil cell tracking in the time-lapse microscopy study lasting for 14 h. Manual tracking (MTrackJ plugging) was performed for each moving neutrophil in each frame. Each time-lapse sequence is composed of 180–210 frames. The video shows representative tracks (color lines) for neutrophils (green, CMFDA). See for instance neutrophils, number 1 and 2, are eventually phagocytosed by a microglial cell (red cell, obtained from a DsRed mouse). (AVI 669 kb)

Online Resource 10. (Figure) Allogenicity does not affect microglia phagocytosis of neutrophils. (PDF 419 kb)

Online Resource 11. (Movie) Time-lapse confocal microscopy study of the phagocytosis of human neutrophils (green) by microglial cells (phase contrast) cultured from a deceased stroke patient. The video covers a period of 12 h in which 720 frames were acquired (one image every minute). (AVI 1962 kb)

Online Resource 12. Flow cytometry of blood from mice treated with control diet or PLEX5622 diet. (PDF 602 kb)

Online Resource 13. Blood cell counts in mice. (PDF 514 kb)

Online Resource 14. Brain infiltrating monocyte/macrophages 4 days post-ischemia. (PDF 337 kb)

## References

[1] Bennett ML, Bennett FC, Liddelow SA, Ajami B, Zamanian JL, Fernhoff NB (2016). New tools for studying microglia in the mouse and human CNS. Proc Natl Acad Sci USA.

[2] Bohlen CJ, Bennett FC, Tucker AF, Collins HY, Mulinyawe SB, Barres BA (2017). Diverse requirements for microglial survival, specification, and function revealed by defined-medium cultures. Neuron.

[3] Brierley JB, Brown AW (1982). The origin of lipid phagocytes in the central nervous system I. The intrinsic microglia. J Comp Neurol.

[4] Brierley JB, Brown AW (1982). The origin of lipid phagocytes in the central nervous system II. The adventitia of blood vessels. J Comp Neurol.

[5] Brown GC, Neher JJ (2012). Eaten alive! Cell death by primary phagocytosis: ‘phagoptosis’. Trends Biochem Sci.

[6] Brown GC, Neher JJ (2014). Microglial phagocytosis of live neurons. Nat Rev Neurosci.

[7] Butovsky O, Jedrychowski MP, Moore CS, Cialic R, Lanser AJ, Gabriely G (2014). Identification of a unique TGF-β-dependent molecular and functional signature in microglia. Nat Neurosci.

[8] Casanova-Acebes M, Pitaval C, Weiss LA, Nombela-Arrieta C, Chèvre R, A-González N (2013). Rhythmic modulation of the hematopoietic niche through neutrophil clearance. Cell.

[9] Chu CH, Wang S, Li CL, Chen SH, Hu CF, Chung YL (2016). Neurons and astroglia govern microglial endotoxin tolerance through macrophage colony-stimulating factor receptor-mediated ERK1/2 signals. Brain Behav Immun.

[10] Chu HX, Kim HA, Lee S, Moore JP, Chan CT, Vinh A (2014). Immune cell infiltration in malignant middle cerebral artery infarction: comparison with transient cerebral ischemia. J Cereb Blood Flow Metab.

[11] Cioce M, Canino C, Goparaju C, Yang H, Carbone M, Pass HI (2014). Autocrine CSF-1R signaling drives mesothelioma chemoresistance via AKT activation. Cell Death Dis.

[12] Conway JG, McDonald B, Parham J, Keith B, Rusnak DW, Shaw E (2005). Inhibition of colony-stimulating-factor-1 signaling in vivo with the orally bioavailable cFMS kinase inhibitor GW2580. Proc Natl Acad Sci USA.

[13] de la Rosa X, Cervera A, Kristoffersen AK, Valdés CP, Varma HM, Justicia C (2014). Mannose-binding lectin promotes local microvascular thrombosis after transient brain ischemia in mice. Stroke.

[14] Denes A, Vidyasagar R, Feng J, Narvainen J, McColl BW, Kauppinen RA (2007). Proliferating resident microglia after focal cerebral ischaemia in mice. J Cereb Blood Flow Metab.

[15] Elmore MR, Najafi AR, Koike MA, Dagher NN, Spangenberg EE, Rice RA (2014). Colony-stimulating factor 1 receptor signaling is necessary for microglia viability, unmasking a microglia progenitor cell in the adult brain. Neuron.

[16] Emerich DF, Dean RL, Bartus RT (2002). The role of leukocytes following cerebral ischemia: pathogenic variable or bystander reaction to emerging infarct?. Exp Neurol.

[17] Enzmann G, Mysiorek C, Gorina R, Cheng YJ, Ghavampour S, Hannocks MJ (2013). The neurovascular unit as a selective barrier to polymorphonuclear granulocyte (PMN) infiltration into the brain after ischemic injury. Acta Neuropathol.

[18] Eyo UB, Miner SA, Ahlers KE, Wu LJ, Dailey ME (2013). P2X7 receptor activation regulates microglial cell death during oxygen-glucose deprivation. Neuropharmacology.

[19] Fan Y, Chen Z, Pathak JL, Carneiro AMD, Chung CY (2018). Differential regulation of adhesion and phagocytosis of resting and activated microglia by dopamine. Front Cell Neurosci.

[20] Fekete R, Cserép C, Lénárt N, Tóth K, Orsolits B, Martinecz B (2018). Microglia control the spread of neurotropic virus infection via P2Y12 signalling and recruit monocytes through P2Y12-independent mechanisms. Acta Neuropathol.

[21] Ferreira TA, Blackman AV, Oyrer J, Jayabal S, Chung AJ, Watt AJ (2014). Neuronal morphometry directly from bitmap images. Nat Methods.

[22] Ganesan LP, Wei G, Pengal RA, Moldovan L, Moldovan N, Ostrowski MC (2004). The serine/threonine kinase Akt Promotes Fc gamma receptor-mediated phagocytosis in murine macrophages through the activation of p70S6 kinase. J Biol Chem.

[23] Garcia JH, Liu KF, Yoshida Y, Lian J, Chen S, del Zoppo GJ (1994). Influx of leukocytes and platelets in an evolving brain infarct (Wistar rat). Am J Pathol.

[24] Gelderblom M, Leypoldt F, Steinbach K, Behrens D, Choe CU, Siler DA (2009). Temporal and spatial dynamics of cerebral immune cell accumulation in stroke. Stroke.

[25] Gidday JM, Gasche YG, Copin JC, Shah AR, Perez RS, Shapiro SD (2005). Leukocyte-derived matrix metalloproteinase-9 mediates blood–brain barrier breakdown and is proinflammatory after transient focal cerebral ischemia. Am J Physiol Heart Circ Physiol.

[26] Hasenberg A, Hasenberg M, Männ L, Neumann F, Borkenstein L, Stecher M (2015). Catchup: a mouse model for imaging-based tracking and modulation of neutrophil granulocytes. Nat Methods.

[27] Heindl S, Gesierich B, Benakis C, Llovera G, Duering M, Liesz A (2018). Automated morphological analysis of microglia after stroke. Front Cell Neurosci.

[28] Haynes SE, Hollopeter G, Yang G, Kurpius D, Dailey ME, Gan WB (2006). The P2Y12 receptor regulates microglial activation by extracellular nucleotides. Nat Neurosci.

[29] Herisson F, Frodermann V, Courties G, Rohde D, Sun Y, Vandoorne K (2018). Direct vascular channels connect skull bone marrow and the brain surface enabling myeloid cell migration. Nat Neurosci.

[30] Hermann DM, Kleinschnitz C, Gunzer M (2018). Implications of polymorphonuclear neutrophils for ischemic stroke and intracerebral hemorrhage: predictive value, pathophysiological consequences and utility as therapeutic target. J Neuroimmunol.

[31] Hermann DM, Kleinschnitz C, Gunzer M (2018). Role of polymorphonuclear neutrophils in the reperfused ischemic brain: insights from cell-type-specific immunodepletion and fluorescence microscopy studies. Ther Adv Neurol Disord.

[32] Hickman SE, Kingery ND, Ohsumi TK, Borowsky ML, Wang LC, Means TK (2013). The microglial sensome revealed by direct RNA sequencing. Nat Neurosci.

[33] Hilla AM, Diekmann H, Fischer D (2017). Microglia are irrelevant for neuronal degeneration and axon regeneration after acute injury. J Neurosci.

[34] Huang LK, Wang MJJ (1995). Image thresholding by minimizing the measure of fuzziness. Pattern Recognit.

[35] Jickling GC, Liu D, Ander BP, Stamova B, Zhan X, Sharp FR (2015). Targeting neutrophils in ischemic stroke: translational insights from experimental studies. J Cereb Blood Flow Metab.

[36] Justicia C, Panés J, Solé S, Cervera A, Deulofeu R, Chamorro A (2003). Neutrophil infiltration increases matrix metalloproteinase-9 in the ischemic brain after occlusion/reperfusion of the middle cerebral artery in rats. J Cereb Blood Flow Metab.

[37] Kierdorf K, Katzmarski N, Haas CA, Prinz M (2013). Bone marrow cell recruitment to the brain in the absence of irradiation or parabiosis bias. PLoS One.

[38] Lalancette-Hebert M, Gowing G, Simard A, Weng YC, Kriz J (2007). Selective ablation of proliferating microglial cells exacerbates ischemic injury in the brain. J Neurosci.

[39] Louis C, Cook AD, Lacey D, Fleetwood AJ, Vlahos R, Anderson GP (2015). Specific contributions of CSF-1 and GM-CSF to the dynamics of the mononuclear phagocyte system. J Immunol.

[40] Li T, Pang S, Yu Y, Wu X, Guo J, Zhang S (2013). Proliferation of parenchymal microglia is the main source of microgliosis after ischaemic stroke. Brain.

[41] Lyons SA, Kettenmann H (1998). Oligodendrocytes and microglia are selectively vulnerable to combined hypoxia and hypoglycemia injury in vitro. J Cereb Blood Flow Metab.

[42] Madisen L, Zwingman TA, Sunkin SM, Oh SW, Zariwala HA, Gu H (2010). A robust and high-throughput Cre reporting and characterization system for the whole mouse brain. Nat Neurosci.

[43] Matsumoto H, Kumon Y, Watanabe H, Ohnishi T, Shudou M, Ii C (2007). Antibodies to CD11b, CD68, and lectin label neutrophils rather than microglia in traumatic and ischemic brain lesions. J Neurosci Res.

[44] McColl BW, Rothwell NJ, Allan SM (2007). Systemic inflammatory stimulus potentiates the acute phase and CXC chemokine responses to experimental stroke and exacerbates brain damage via interleukin-1- and neutrophil-dependent mechanisms. J Neurosci.

[45] Meijering E, Dzyubachyk O, Smal I (2012). Methods for cell and particle tracking. Methods Enzymol.

[46] Miró-Mur F, Pérez-de-Puig I, Ferrer-Ferrer M, Urra X, Justicia C, Chamorro A (2016). Immature monocytes recruited to the ischemic mouse brain differentiate into macrophages with features of alternative activation. Brain Behav Immun.

[47] Mitrasinovic OM, Grattan A, Robinson CC, Lapustea NB, Poon C, Ryan H (2005). Microglia overexpressing the macrophage colony-stimulating factor receptor are neuroprotective in a microglial-hippocampal organotypic coculture system. J Neurosci.

[48] Neher JJ, Emmrich JV, Fricker M, Mander PK, Théry C, Brown GC (2013). Phagocytosis executes delayed neuronal death after focal brain ischemia. Proc Natl Acad Sci USA.

[49] Neher JJ, Neniskyte U, Zhao JW, Bal-Price A, Tolkovsky AM, Brown GC (2011). Inhibition of microglial phagocytosis is sufficient to prevent inflammatory neuronal death. J Immunol.

[50] Neumann J, Henneberg S, von Kenne S, Nolte N, Müller AJ, Schraven B (2018). Beware the intruder: real time observation of infiltrated neutrophils and neutrophil-Microglia interaction during stroke in vivo. PLoS One.

[51] Neumann J, Riek-Burchardt M, Herz J, Doeppner TR, König R, Hütten H (2015). Very-late-antigen-4 (VLA-4)-mediated brain invasion by neutrophils leads to interactions with microglia, increased ischemic injury and impaired behavior in experimental stroke. Acta Neuropathol.

[52] Neumann J, Sauerzweig S, Rönicke R, Gunzer F, Dinkel K, Ullrich O (2008). Microglia cells protect neurons by direct engulfment of invading neutrophil granulocytes: a new mechanism of CNS immune privilege. J Neurosci.

[53] Nourshargh S, Alon R (2014). Leukocyte migration into inflamed tissues. Immunity.

[54] Olmos-Alonso A, Schetters ST, Sri S, Askew K, Mancuso R, Vargas-Caballero M (2016). Pharmacological targeting of CSF1R inhibits microglial proliferation and prevents the progression of Alzheimer’s-like pathology. Brain.

[55] Perez-de-Puig I, Miró-Mur F, Ferrer-Ferrer M, Gelpi E, Pedragosa J, Justicia C (2015). Neutrophil recruitment to the brain in mouse and human ischemic stroke. Acta Neuropathol.

[56] Price CJ, Menon DK, Peters AM, Ballinger JR, Barber RW, Balan KK (2004). Cerebral neutrophil recruitment, histology, and outcome in acute ischemic stroke: an imaging-based study. Stroke.

[57] Rosell A, Cuadrado E, Ortega-Aznar A, Hernández-Guillamon M, Lo EH, Montaner J (2008). MMP-9-positive neutrophil infiltration is associated to blood–brain barrier breakdown and basal lamina type IV collagen degradation during hemorrhagic transformation after human ischemic stroke. Stroke.

[58] Sampaio NG, Yu W, Cox D, Wyckoff J, Condeelis J, Stanley ER (2011). Phosphorylation of CSF-1R Y721 mediates its association with PI3K to regulate macrophage motility and enhancement of tumor cell invasion. J Cell Sci.

[59] Sasaki Y, Hoshi M, Akazawa C, Nakamura Y, Tsuzuki H, Inoue K (2003). Selective expression of Gi/o-coupled ATP receptor P2Y12 in microglia in rat brain. Glia.

[60] Satoh J, Asahina N, Kitano S, Kino Y (2014). A comprehensive profile of ChIP-Seq-based PU.1/Spi1 target genes in microglia. Gene Regul Syst Biol.

[61] Segel GB, Halterman MW, Lichtman MA (2011). The paradox of the neutrophil’s role in tissue injury. J Leukoc Biol.

[62] Smith AM, Gibbons HM, Oldfield RL, Bergin PM, Mee EW, Curtis MA (2013). M-CSF increases proliferation and phagocytosis while modulating receptor and transcription factor expression in adult human microglia. J Neuroinflamm.

[63] Song J, Zhang X, Buscher K, Wang Y, Wang H, Di Russo J (2017). Endothelial basement membrane laminin 511 contributes to endothelial junctional tightness and thereby inhibits leukocyte transmigration. Cell Rep.

[64] Stowe AM, Adair-Kirk TL, Gonzales ER, Perez RS, Shah AR, Park TS (2009). Neutrophil elastase and neurovascular injury following focal stroke and reperfusion. Neurobiol Dis.

[65] Szalay G, Martinecz B, Lénárt N, Környei Z, Orsolits B, Judák L (2016). Microglia protect against brain injury and their selective elimination dysregulates neuronal network activity after stroke. Nat Commun.

[66] Turner RJ, Sharp FR (2016). Implications of MMP9 for blood brain barrier disruption and hemorrhagic transformation following ischemic stroke. Front Cell Neurosci.

[67] Vergadi E, Ieronymaki E, Lyroni K, Vaporidi K, Tsatsanis C (2017). Akt signaling pathway in macrophage activation and M1/M2 polarization. J Immunol.

[68] Wagner T, Lipinski H-G (2013). IJBlob: an ImageJ library for connected component analysis and shape analysis. J Open Res Softw.

[69] Waisman A, Ginhoux F, Greter M, Bruttger J (2015). Homeostasis of microglia in the adult brain: review of novel microglia depletion systems. Trends Immunol.

[70] Wheeler DL, Sariol A, Meyerholz DK, Perlman S (2018). Microglia are required for protection against lethal coronavirus encephalitis in mice. J Clin Investig.

[71] Weinhard L, di Bartolomei G, Bolasco G, Machado P, Schieber NL, Neniskyte U (2018). Microglia remodel synapses by presynaptic trogocytosis and spine head filopodia induction. Nat Commun.

[72] Yang Y, Rosenberg GA (2011). Blood–brain barrier breakdown in acute and chronic cerebrovascular disease. Stroke.

[73] Yenari MA, Giffard RG (2001). Ischemic vulnerability of primary murine microglial cultures. Neurosci Lett.

[74] Yona S, Kim KW, Wolf Y, Mildner A, Varol D, Breker M (2013). Fate mapping reveals origins and dynamics of monocytes and tissue macrophages under homeostasis. Immunity.

[75] Zec K, Volke J, Vijitha N, Thiebes S, Gunzer M, Kurts C (2016). Neutrophil migration into the infected uroepithelium is regulated by the crosstalk between resident and helper macrophages. Pathogens.

[76] Zrzavy T, Machado-Santos J, Christine S, Baumgartner C, Weiner HL, Butovsky O (2017). Dominant role of microglial and macrophage innate immune responses in human ischemic infarcts. Brain Pathol.

[77] Yu W, Chen J, Xiong Y, Pixley FJ, Dai XM, Yeung YG (2008). CSF-1 receptor structure/function in MacCsf1r^−/−^ macrophages: regulation of proliferation, differentiation, and morphology. J Leukoc Biol.

