# Supplementary material for: Microglial cell loss after ischemic stroke favors brain neutrophil accumulation
Source: Acta Neuropathol. 2018 Dec 22;137(2):321–41. doi: 10.1007/s00401-018-1954-4 (PMC6513908; doi:10.1007/s00401-018-1954-4)
Supplement: Supplementary file 1 — Online Resource 1. (Table) Clinical and radiological characteristics of the stroke patients. (PDF 84 kb) [file 401_2018_1954_MOESM1_ESM.pdf]

Online Resource 1

Microglial cell loss after ischemic stroke favors brain neutrophil accumulation

ACTA NEUROPATHOLOGICA

Amaia Otxoa-de-Amezaga[1,2], Francesc Miró-Mur[2], Jordi Pedragosa[1,2], Mattia Gallizioli[1,2], Carles Justicia[1,2], Núria Gaja-Capdevila[1], Francisca Ruíz-Jaen[1,2], Angélica Salas-Perdomo[1,2], Anna Bosch[3], Maria Calvo[3], Leonardo Marquez-Kisinousky[1], Adam Denes[4], Matthias Gunzer[5], Anna M. Planas[1,2]

Author Affiliations

[1] Department of Brain Ischemia and Neurodegeneration, Institut d'Investigacions Biomèdiques de Barcelona (IIBB)-Consejo Superior de Investigaciones Científicas (CSIC), Barcelona, Spain

[2] Institut d'Investigacions Biomèdiques August Pi i Sunyer (IDIBAPS), Barcelona, Spain

[3] Serveis Científico-Tècnics de Universitat de Barcelona, Campus Casanova, Barcelona, Spain

[4] Laboratory of Neuroimmunology, Institute of Experimental Medicine, Hungarian Academy of Sciences, Budapest, Hungary

[5] Institute for Experimental Immunology and Imaging, University Hospital, University Duisburg–Essen, Essen, Germany

* Corresponding author:

Anna M. Planas
IIBB-CSIC, IDIBAPS
Rosselló 161, planta 6, 08036-Barcelona, Spain
Tel:+34-933638327 Fax: +34-933638301
e-mail: anna.planas@iibb.csic.es

Online Resource 1. Clinical and radiological characteristics of the stroke patients.

Case	Age	Gender	Admission NIHSS score	Vascular territory	Intracranial occlusion site	Acute revascula-rization therapy	Hemorrhagic transformation	Etiology	Stroke onset to exitus time (days)	Exitus to necropsy time (hours)
1	88	Woman	19	Carotid	Right and left M1	Systemic thrombolysis	No	Cardioembolic	5	4
2	79	Man	4	Left MCA infarct	Left M2	None	Parenchymal hematoma type 2	Large vessel disease	5	4
3	86	Woman	1	Vertebro-basilar infarct	Basilar artery	None	No	Cardioembolic	3	3
4	89	Woman	9	Vertebro-basilar infarct	No vessel imaging	None	No	Cardioembolic	1	3
5	63	Woman	20	Right MCA infarct	Right M1	Mechanical thrombectomy	No	Undetermined	2	2
6	86	Woman	20	Left MCA infarct	Left M1	None	No	Cardioembolic	6	2

NIHSS: National Institutes of Health Stroke Scale; MCA: middle cerebral artery; M1: M1 segment of the middle cerebral artery; M2: M2 segment of the middle cerebral artery.
