# Supplementary material for: Microglial cell loss after ischemic stroke favors brain neutrophil accumulation
Source: Acta Neuropathol. 2018 Dec 22;137(2):321–41. doi: 10.1007/s00401-018-1954-4 (PMC6513908; doi:10.1007/s00401-018-1954-4)
Supplement: Supplementary file 2 — Online Resource 2. (Figure) Flow cytometry analysis of blood and brain tissue of DsRed chimeric mice two month after bone marrow transfer of DsRed cells. (PDF 1258 kb) [file 401_2018_1954_MOESM2_ESM.pdf]

Online Resource 2

Microglial cell loss after ischemic stroke favors brain neutrophil accumulation

ACTA NEUROPATHOLOGICA

Amaia Otxoa-de-Amezaga[1,2], Francesc Miró-Mur[2], Jordi Pedragosa[1,2], Mattia Gallizioli[1,2], Carles Justicia[1,2], Núria Gaja-Capdevila[1], Francisca Ruíz-Jaen[1,2], Angélica Salas-Perdomo[1,2], Anna Bosch[3], Maria Calvo[3], Leonardo Marquez-Kisinousky[1], Adam Denes[4], Matthias Gunzer[5], Anna M. Planas[1,2]

Author Affiliations

[1] Department of Brain Ischemia and Neurodegeneration, Institut d'Investigacions Biomèdiques de Barcelona (IIBB)-Consejo Superior de Investigaciones Científicas (CSIC), Barcelona, Spain

[2] Institut d'Investigacions Biomèdiques August Pi i Sunyer (IDIBAPS), Barcelona, Spain

[3] Serveis Científico-Tècnics de Universitat de Barcelona, Campus Casanova, Barcelona, Spain

[4] Laboratory of Neuroimmunology, Institute of Experimental Medicine, Hungarian Academy of Sciences, Budapest, Hungary

[5] Institute for Experimental Immunology and Imaging, University Hospital, University Duisburg–Essen, Essen, Germany

* Corresponding author:

Anna M. Planas
IIBB-CSIC, IDIBAPS
Rosselló 161, planta 6, 08036-Barcelona, Spain
Tel:+34-933638327 Fax: +34-933638301
e-mail: anna.planas@iibb.csic.es

Online Resource 2

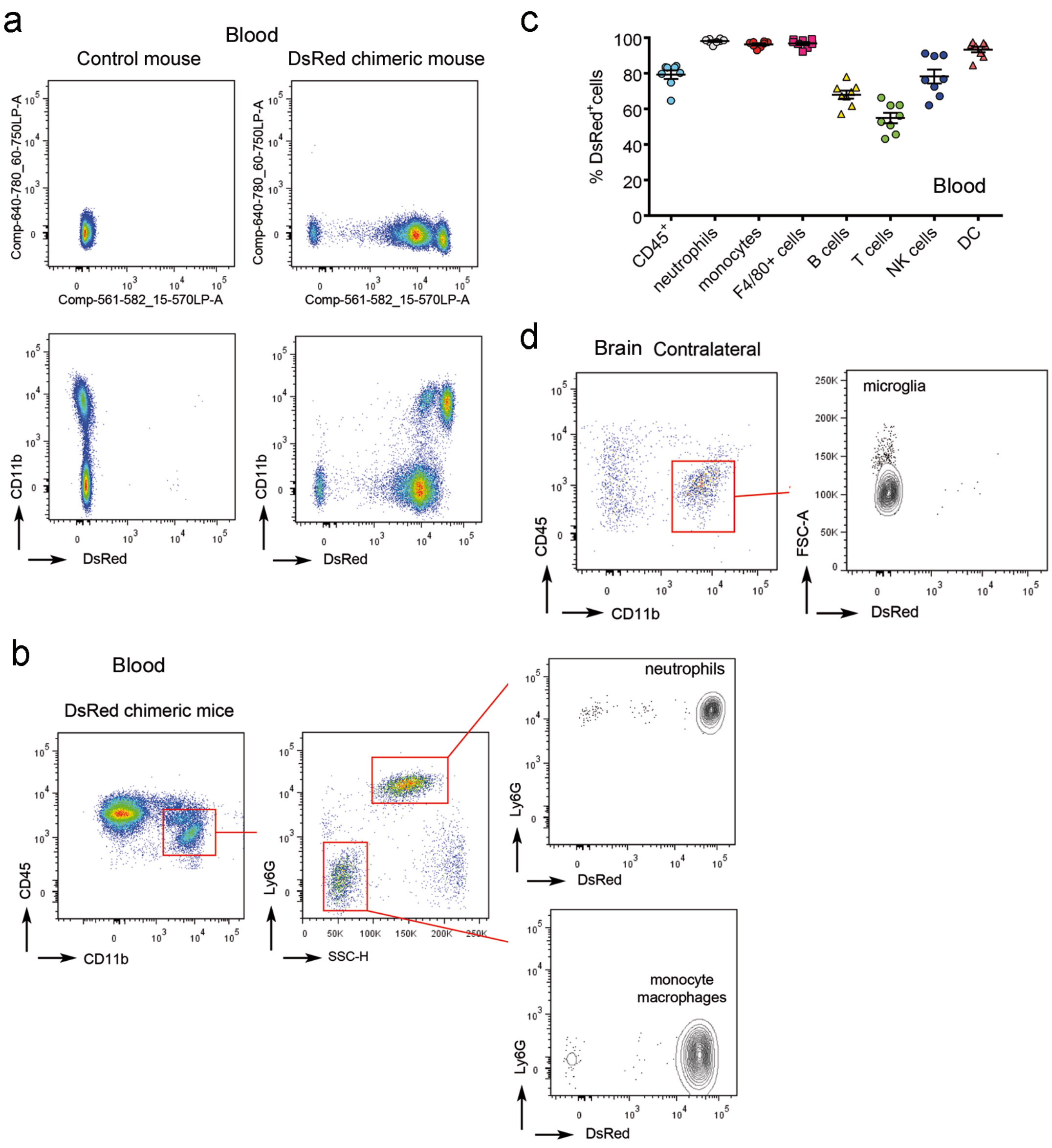

Online Resource 2. Flow cytometry analysis of blood and brain tissue of DsRed chimeric mice two month after bone marrow transfer of DsRed cells. a) Assessment of blood chimerism by flow cytometry showing the presence of DsRed cells in the chimeric mice and absence in control wild type mice. **b,c**) Blood myeloid cells show a high degree of chimerism (n=8 mice). **d**) Analysis of the contralateral brain hemispheres of DsRed chimeric mice 4 days after MCAo. DsRed cells were not found in the contralateral hemisphere. CD45^{dim}CD11b^{dim} microglial cells are DsRed^{-}. Plots are representative of n=5 ischemic mice.
