# Supplementary material for: Microglial cell loss after ischemic stroke favors brain neutrophil accumulation
Source: Acta Neuropathol. 2018 Dec 22;137(2):321–41. doi: 10.1007/s00401-018-1954-4 (PMC6513908; doi:10.1007/s00401-018-1954-4)
Supplement: Supplementary file 3 — Online Resource 3. (Figure) Flow cytometry analysis of tdT+ immune cells in CX3CR1cre/ert2 :Rosa26-tdT mice. (PDF 3076 kb) [file 401_2018_1954_MOESM3_ESM.pdf]

Online Resource 3

Microglial cell loss after ischemic stroke favors brain neutrophil accumulation

ACTA NEUROPATHOLOGICA

Amaia Otxoa-de-Amezaga[1,2], Francesc Miró-Mur[2], Jordi Pedragosa[1,2], Mattia Gallizioli[1,2], Carles Justicia[1,2], Núria Gaja-Capdevila[1], Francisca Ruíz-Jaen[1,2], Angélica Salas-Perdomo[1,2], Anna Bosch[3], Maria Calvo[3], Leonardo Marquez-Kisinousky[1], Adam Denes[4], Matthias Gunzer[5], Anna M. Planas[1,2]

Author Affiliations

[1] Department of Brain Ischemia and Neurodegeneration, Institut d'Investigacions Biomèdiques de Barcelona (IIBB)-Consejo Superior de Investigaciones Científicas (CSIC), Barcelona, Spain

[2] Institut d'Investigacions Biomèdiques August Pi i Sunyer (IDIBAPS), Barcelona, Spain

[3] Serveis Científico-Tècnics de Universitat de Barcelona, Campus Casanova, Barcelona, Spain

[4] Laboratory of Neuroimmunology, Institute of Experimental Medicine, Hungarian Academy of Sciences, Budapest, Hungary

[5] Institute for Experimental Immunology and Imaging, University Hospital, University Duisburg–Essen, Essen, Germany

* Corresponding author:

Anna M. Planas
IIBB-CSIC, IDIBAPS
Rosselló 161, planta 6, 08036-Barcelona, Spain
Tel:+34-933638327 Fax: +34-933638301
e-mail: anna.planas@iibb.csic.es

Online Resource 3

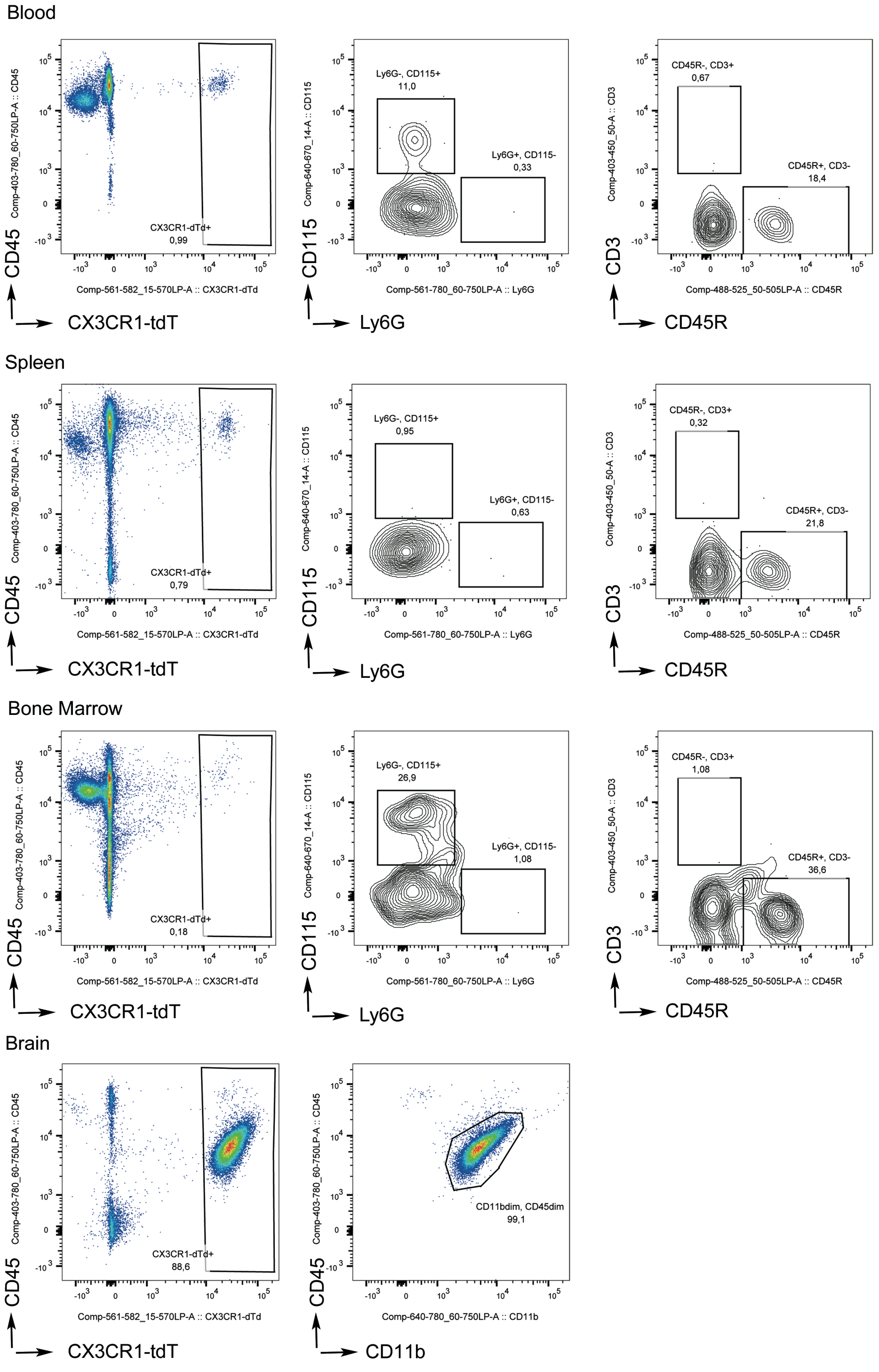

Online Resource 3. Flow cytometry analysis of tdT$^+$ immune cells in CX3CR1$^{cre/ert2}$:Rosa26-tdT mice. Mice (n=5) received tamoxifen treatment and were studied 4 weeks later to allow for clearing of possible tdT$^+$ peripheral cells. Most microglial cells were tdT$^+$ (99%), whereas comparatively only a few peripheral leukocytes were tdT$^+$. We observed some tdT$^+$ B cells (CD45R$^+$) in blood, spleen and bone marrow, and some tdT$^+$ monocytes (CD115$^+$) in blood and bone marrow. In contrast, T cells and neutrophils were tdT$^-$.
