# Supplementary material for: Microglial cell loss after ischemic stroke favors brain neutrophil accumulation
Source: Acta Neuropathol. 2018 Dec 22;137(2):321–41. doi: 10.1007/s00401-018-1954-4 (PMC6513908; doi:10.1007/s00401-018-1954-4)
Supplement: Supplementary file 4 — Online Resource 4. (Figure) Morphological analysis of microglia. (PDF 341 kb) [file 401_2018_1954_MOESM4_ESM.pdf]

## Online Resource 4

# Microglial cell loss after ischemic stroke favors brain neutrophil accumulation

**ACTA NEUROPATHOLOGICA**

Amaia Otxoa-de-Amezaga[1,2], Francesc Miró-Mur[2], Jordi Pedragosa[1,2], Mattia Gallizioli[1,2], Carles Justicia[1,2], Núria Gaja-Capdevila[1], Francisca Ruíz-Jaen[1,2], Angélica Salas-Perdomo[1,2], Anna Bosch[3], Maria Calvo[3], Leonardo Marquez-Kisinousky[1], Adam Denes[4], Matthias Gunzer[5], Anna M. Planas[1,2]

**Author Affiliations**

[1] Department of Brain Ischemia and Neurodegeneration, Institut d'Investigacions Biomèdiques de Barcelona (IIBB)-Consejo Superior de Investigaciones Científicas (CSIC), Barcelona, Spain

[2] Institut d'Investigacions Biomèdiques August Pi i Sunyer (IDIBAPS), Barcelona, Spain

[3] Serveis Científico-Tècnics de Universitat de Barcelona, Campus Casanova, Barcelona, Spain

[4] Laboratory of Neuroimmunology, Institute of Experimental Medicine, Hungarian Academy of Sciences, Budapest, Hungary

[5] Institute for Experimental Immunology and Imaging, University Hospital, University Duisburg–Essen, Essen, Germany

* Corresponding author:

Anna M. Planas
IIBB-CSIC, IDIBAPS
Rosselló 161, planta 6, 08036-Barcelona, Spain
Tel:+34-933638327 Fax: +34-933638301
e-mail: anna.planas@iibb.csic.es

Online Resource 4

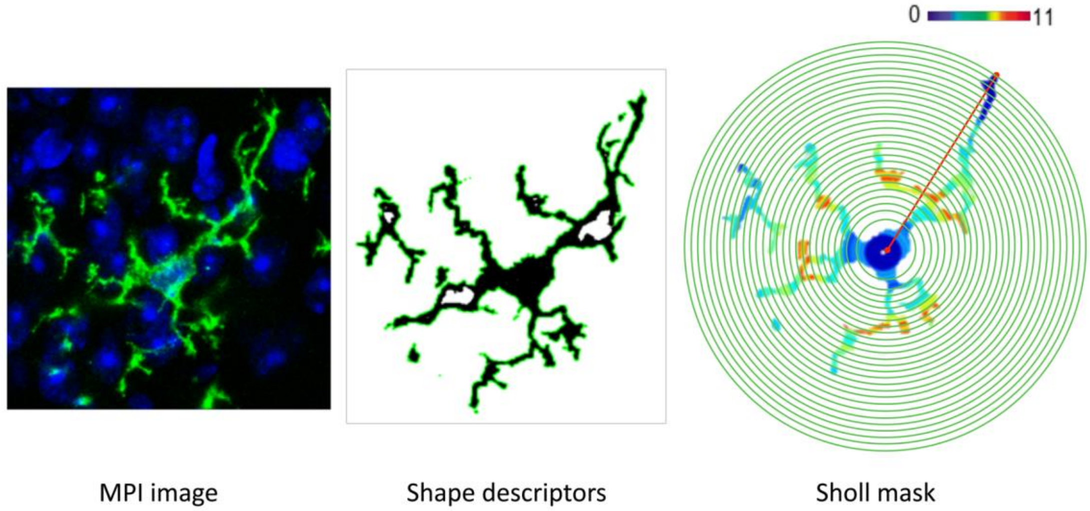


**Online Resource 4. Morphological analysis of microglia.** We used shape descriptors and Sholl analysis as described in Methods. After the segmentation, the ramification index is measured by Sholl analysis. The intersection mask  (see image on the right hand side) is generated by indicating microglial arborization (ramification index) with a color scale according to its Sholl profile. This Sholl mask is a representation of the analyzed cell where intersection counts are used as pixel intensities. The red color in the example shown here for one microglial cell corresponds to 11 intersections.
