# Supplementary material for: Microglial cell loss after ischemic stroke favors brain neutrophil accumulation
Source: Acta Neuropathol. 2018 Dec 22;137(2):321–41. doi: 10.1007/s00401-018-1954-4 (PMC6513908; doi:10.1007/s00401-018-1954-4)
Supplement: Supplementary file 10 — Online Resource 10. (Figure) Allogenicity does not affect microglia phagocytosis of neutrophils. (PDF 419 kb) [file 401_2018_1954_MOESM10_ESM.pdf]

Online Resource 10

Microglial cell loss after ischemic stroke favors brain neutrophil accumulation

ACTA NEUROPATHOLOGICA

Amaia Otxoa-de-Amezaga[1,2], Francesc Miró-Mur[2], Jordi Pedragosa[1,2], Mattia Gallizioli[1,2], Carles Justicia[1,2], Núria Gaja-Capdevila[1], Francisca Ruíz-Jaen[1,2], Angélica Salas-Perdomo[1,2], Anna Bosch[3], Maria Calvo[3], Leonardo Marquez-Kisinousky[1], Adam Denes[4], Matthias Gunzer[5], Anna M. Planas[1,2]

Author Affiliations

[1] Department of Brain Ischemia and Neurodegeneration, Institut d'Investigacions Biomèdiques de Barcelona (IIBB)-Consejo Superior de Investigaciones Científicas (CSIC), Barcelona, Spain

[2] Institut d'Investigacions Biomèdiques August Pi i Sunyer (IDIBAPS), Barcelona, Spain

[3] Serveis Científico-Tècnics de Universitat de Barcelona, Campus Casanova, Barcelona, Spain

[4] Laboratory of Neuroimmunology, Institute of Experimental Medicine, Hungarian Academy of Sciences, Budapest, Hungary

[5] Institute for Experimental Immunology and Imaging, University Hospital, University Duisburg–Essen, Essen, Germany

* Corresponding author:

Anna M. Planas
IIBB-CSIC, IDIBAPS
Rosselló 161, planta 6, 08036-Barcelona, Spain
Tel:+34-933638327 Fax: +34-933638301
e-mail: anna.planas@iibb.csic.es

Online Resource 10

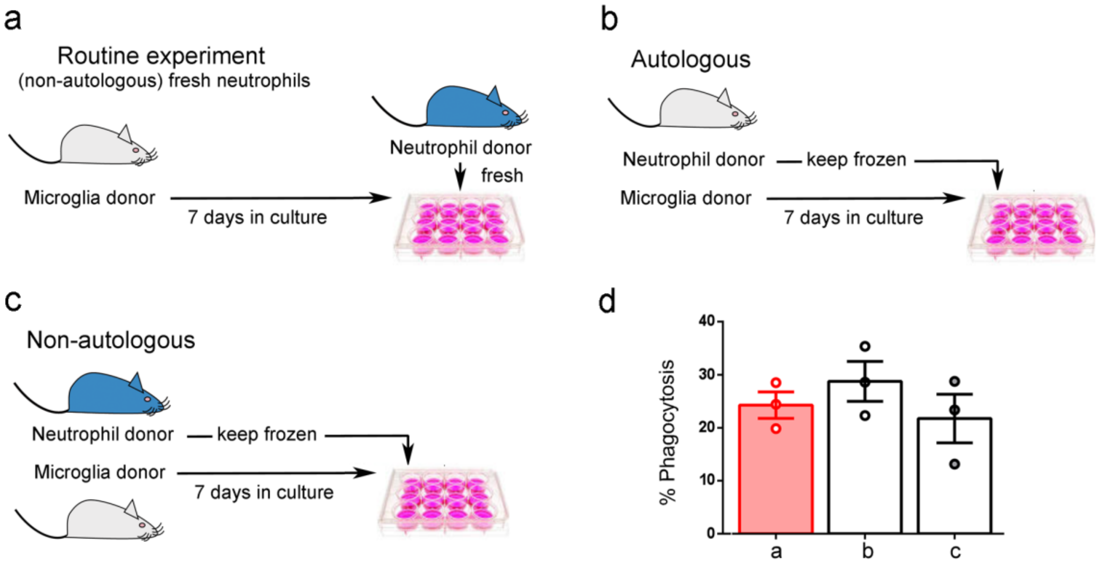

Online Resource 10. Allogenicity does not affect microglia phagocytosis of neutrophils. We performed experiments to control for the possibility that allogenicity contributed to phagocytosis of neutrophils by microglia. **a**) Our routine experiments were performed by obtaining microglia from a donor mouse and culturing the cells for 7 days. The day of the experiment, we obtained neutrophils from the bone marrow of another mouse and fresh neutrophils were added to the microglia culture for time-lapse microscopy studies of phagocytosis. **b**) To use an autologous cellular system, we used a single donor mouse to obtain microglia from the brain and neutrophils from the bone marrow of the same animal. Microglia was cultured for 7 days whereas neutrophils were cryoprotected and frozen until the day of the experiment. **c**) For a parallel non-autologous system, we obtained microglia from the brain of a donor mouse and used a different mouse to obtain bone marrow neutrophils on the same day. Microglia was cultured for 7 days whereas neutrophils were cryoprotected and frozen until the day of the experiment. **d**) Results showed no significant differences in the % of microglia phagocytosing neutrophils between the different experimental groups. n=3 independent experiments per group.
