# Supplementary material for: Microglial cell loss after ischemic stroke favors brain neutrophil accumulation
Source: Acta Neuropathol. 2018 Dec 22;137(2):321–41. doi: 10.1007/s00401-018-1954-4 (PMC6513908; doi:10.1007/s00401-018-1954-4)
Supplement: Supplementary file 13 — Online Resource 13. Blood cell counts in mice. (PDF 514 kb) [file 401_2018_1954_MOESM13_ESM.pdf]

Online Resource 13

Microglial cell loss after ischemic stroke favors brain neutrophil accumulation

ACTA NEUROPATHOLOGICA

Amaia Otxoa-de-Amezaga[1,2], Francesc Miró-Mur[2], Jordi Pedragosa[1,2], Mattia Gallizioli[1,2], Carles Justicia[1,2], Núria Gaja-Capdevila[1], Francisca Ruíz-Jaen[1,2], Angélica Salas-Perdomo[1,2], Anna Bosch[3], Maria Calvo[3], Leonardo Marquez-Kisinousky[1], Adam Denes[4], Matthias Gunzer[5], Anna M. Planas[1,2]

Author Affiliations

[1] Department of Brain Ischemia and Neurodegeneration, Institut d'Investigacions Biomèdiques de Barcelona (IIBB)-Consejo Superior de Investigaciones Científicas (CSIC), Barcelona, Spain

[2] Institut d'Investigacions Biomèdiques August Pi i Sunyer (IDIBAPS), Barcelona, Spain

[3] Serveis Científico-Tècnics de Universitat de Barcelona, Campus Casanova, Barcelona, Spain

[4] Laboratory of Neuroimmunology, Institute of Experimental Medicine, Hungarian Academy of Sciences, Budapest, Hungary

[5] Institute for Experimental Immunology and Imaging, University Hospital, University Duisburg–Essen, Essen, Germany

* Corresponding author:

Anna M. Planas
IIBB-CSIC, IDIBAPS
Rosselló 161, planta 6, 08036-Barcelona, Spain
Tel:+34-933638327 Fax: +34-933638301
e-mail: anna.planas@iibb.csic.es

Online Resource 13

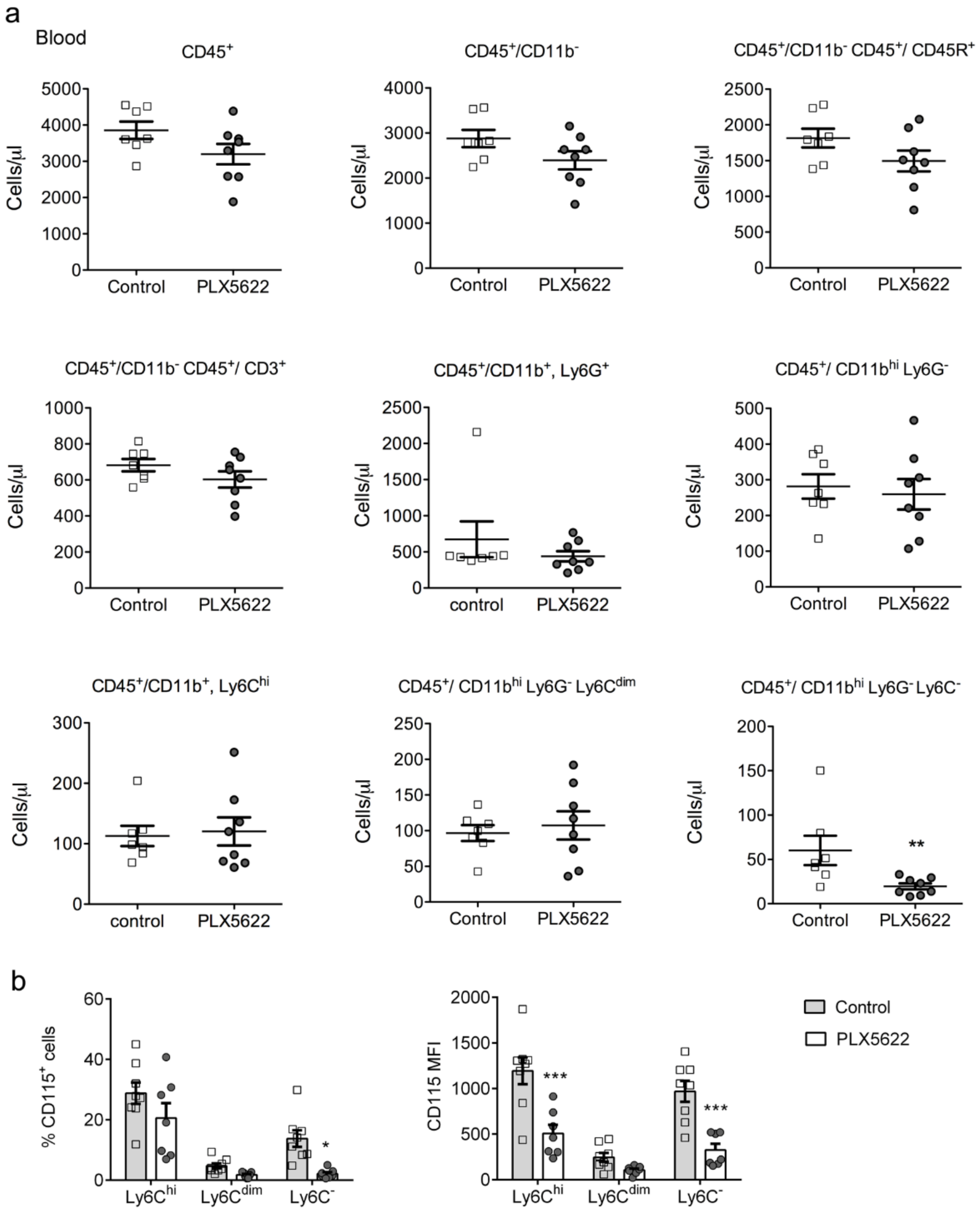

Online Resource 13. Blood cell counts in mice. Mice received control diet (n=7) or PLX5622 (n=8) diet for 3 weeks prior to ischemia and were studied 4 days post-ischemia. a) Amongst the different leukocyte populations, we only found statistically significant differences in a subset of Ly6C negative monocytes that decreased in mice receiving PLX5622 (Mann-Whitney test, $^{**}p=0.006$). b) Analysis of expression of CD115 (CSF1R) within the Monocyte subpopulations defined according to the level of Ly6C expression. The percentage of $CD115^+$ cells decreased within the $Ly6C^-$ population (Two-way ANOVA by treatment and monocyte subset with subject matching followed by the Bonferroni test, $^{*}p<0.05$). The mean fluorescence intensity (MFI) of CD115 decreased in the populations of $Ly6C^{hi}$ and $Ly6C^-$ monocytes (Two-way ANOVA by treatment and monocyte subset with subject matching followed by the Bonferroni test, $^{***}p<0.001$). Bars represent the mean ±SEM and symbols correspond to values of the different mice.
