# Supplementary material for: Microglial cell loss after ischemic stroke favors brain neutrophil accumulation
Source: Acta Neuropathol. 2018 Dec 22;137(2):321–41. doi: 10.1007/s00401-018-1954-4 (PMC6513908; doi:10.1007/s00401-018-1954-4)
Supplement: Supplementary file 14 — Online Resource 14. Brain infiltrating monocyte/macrophages 4 days post-ischemia. (PDF 337 kb) [file 401_2018_1954_MOESM14_ESM.pdf]

Online Resource 14

Microglial cell loss after ischemic stroke favors brain neutrophil accumulation

ACTA NEUROPATHOLOGICA

Amaia Otxoa-de-Amezaga[1,2], Francesc Miró-Mur[2], Jordi Pedragosa[1,2], Mattia Gallizioli[1,2], Carles Justicia[1,2], Núria Gaja-Capdevila[1], Francisca Ruíz-Jaen[1,2], Angélica Salas-Perdomo[1,2], Anna Bosch[3], Maria Calvo[3], Leonardo Marquez-Kisinousky[1], Adam Denes[4], Matthias Gunzer[5], Anna M. Planas[1,2]

Author Affiliations

[1] Department of Brain Ischemia and Neurodegeneration, Institut d'Investigacions Biomèdiques de Barcelona (IIBB)-Consejo Superior de Investigaciones Científicas (CSIC), Barcelona, Spain

[2] Institut d'Investigacions Biomèdiques August Pi i Sunyer (IDIBAPS), Barcelona, Spain

[3] Serveis Científico-Tècnics de Universitat de Barcelona, Campus Casanova, Barcelona, Spain

[4] Laboratory of Neuroimmunology, Institute of Experimental Medicine, Hungarian Academy of Sciences, Budapest, Hungary

[5] Institute for Experimental Immunology and Imaging, University Hospital, University Duisburg–Essen, Essen, Germany

* Corresponding author:

Anna M. Planas
IIBB-CSIC, IDIBAPS
Rosselló 161, planta 6, 08036-Barcelona, Spain
Tel:+34-933638327 Fax: +34-933638301
e-mail: anna.planas@iibb.csic.es

Online Resource 14

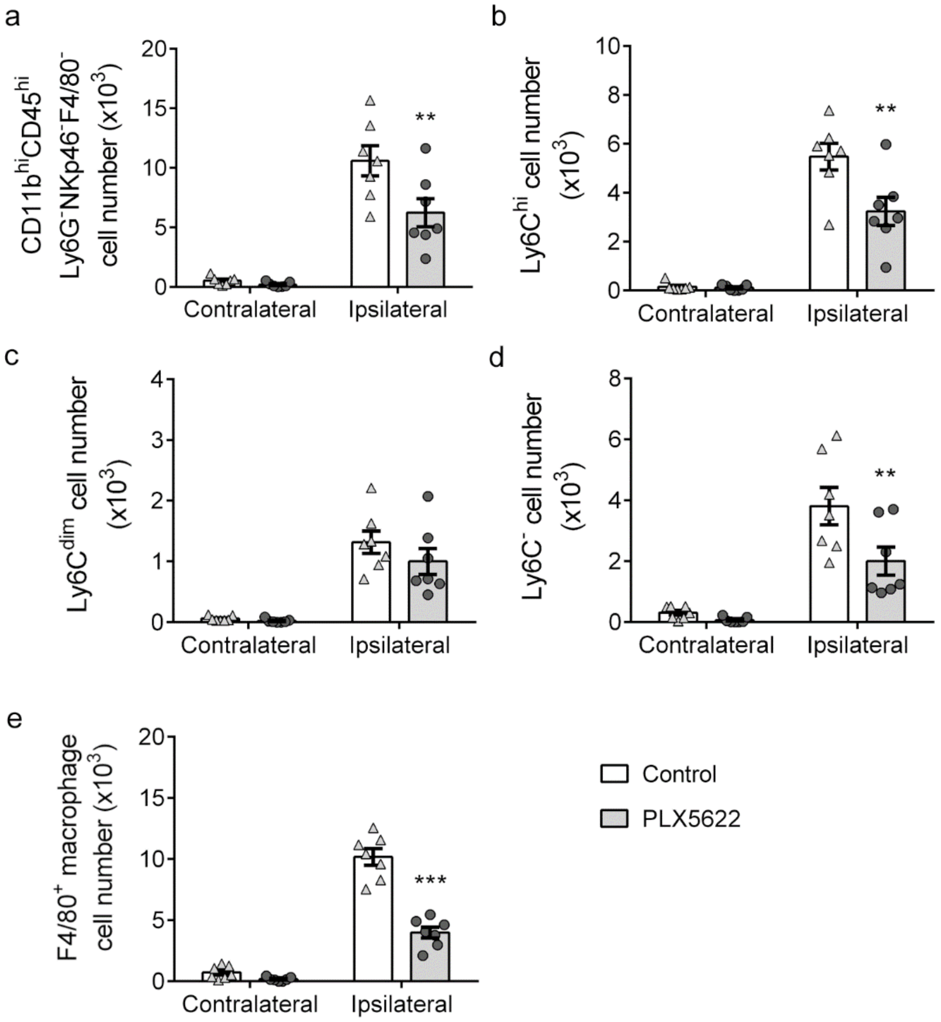

Online Resource 14. Brain infiltrating monocyte/macrophages 4 days post-ischemia. Mice received control diet or PLX5622 diet (n=7 per group) for 3 weeks prior to induction of ischemia and the contralateral and ipsilateral (ischemic) brain hemispheres were studied at day 4 post-ischemia by flow cytometry. a) PLX5622 reduced the number of monocytes ($CD11b^{hi}$ $CD45^{hi}$ $Ly6G^{-}NKp46^{-}$ $F4/80^{-}$ cells) infiltrating the ipsilateral brain hemisphere. b-d) Amongst them, the subset of $Ly6C^{dim}$ monocytes was not affected by the treatment (c), but the numbers of $Ly6C^{hi}$ (b) and $Ly6C^{-}$ (d) monocytes decreased after PLEX5622. d) $F4/80^{+}$ macrophages also decreased in mice receiveing the PLX5622 diet. Two-way ANOVA by treatment and brain hemisphere with subject matching followed by the Bonferroni test, ** $p<0.01$, *** $p<0.001$. Bars show the mean ± SEM and points indicate the values of individual mice.
